# Bayesian Reconstruction of Disease Outbreaks by Combining Epidemiologic and Genomic Data

**DOI:** 10.1371/journal.pcbi.1003457

**Published:** 2014-01-23

**Authors:** Thibaut Jombart, Anne Cori, Xavier Didelot, Simon Cauchemez, Christophe Fraser, Neil Ferguson

**Affiliations:** MRC Centre for Outbreak Analysis and Modelling, Department of Infectious Disease Epidemiology, School of Public Health, Imperial College London, London, United Kingdom; University of New South Wales, Australia

## Abstract

Recent years have seen progress in the development of statistically rigorous frameworks to infer outbreak transmission trees (“who infected whom”) from epidemiological and genetic data. Making use of pathogen genome sequences in such analyses remains a challenge, however, with a variety of heuristic approaches having been explored to date. We introduce a statistical method exploiting both pathogen sequences and collection dates to unravel the dynamics of densely sampled outbreaks. Our approach identifies likely transmission events and infers dates of infections, unobserved cases and separate introductions of the disease. It also proves useful for inferring numbers of secondary infections and identifying heterogeneous infectivity and super-spreaders. After testing our approach using simulations, we illustrate the method with the analysis of the beginning of the 2003 Singaporean outbreak of Severe Acute Respiratory Syndrome (SARS), providing new insights into the early stage of this epidemic. Our approach is the first tool for disease outbreak reconstruction from genetic data widely available as free software, the R package *outbreaker*. It is applicable to various densely sampled epidemics, and improves previous approaches by detecting unobserved and imported cases, as well as allowing multiple introductions of the pathogen. Because of its generality, we believe this method will become a tool of choice for the analysis of densely sampled disease outbreaks, and will form a rigorous framework for subsequent methodological developments.

## Author Summary

Understanding how infectious diseases are transmitted from one individual to another is essential for designing containment strategies and epidemic prevention. Recently, the reconstruction of transmission trees (“who infected whom”) has been revolutionized by the availability of pathogen genome sequences. Exploiting this information remains a challenge, however, with a variety of heuristic approaches having been explored to date. Here, we introduce a new method which uses both pathogen DNA and collection dates to gain insights into transmission events, and detect unobserved cases and separate introductions of the disease. Our approach is also useful for identifying super-spreaders, i.e., cases which caused many subsequent infections. After testing our method using simulations, we use it to gain new insights into the beginning of the 2003 Singaporean outbreak of Severe Acute Respiratory Syndrome (SARS). Our approach is applicable to a wide range of diseases and available in a free software package called *outbreaker*.

This is a *PLOS Computational Biology* Methods article.

## Introduction

Statistical methods for analyzing detailed epidemiological data collected during infectious disease outbreaks have seen rapid development in recent years [1], [2], [3], [4], [5], [6], [7], [8]. These methods probabilistically reconstruct likely transmission links between cases using data on the timing of symptoms and, where available, contact tracing data or other proximity information. The resulting transmission trees allow estimation of the number of secondary infections generated by each case, and thus of the transmission intensity (characterized by the reproduction number, *R*) over time. Pathogen genetic sequence data provides valuable additional information on potential transmission links between cases in a disease outbreak, particularly when reliable contact tracing data is not available. Indeed, using sequence data alone to estimate transmission rates during epidemics is increasingly frequent [9], [10], [11], [12], [13], [14], [15], [16], [17], [18], [19]. As genetic sequences can now be obtained nearly in real-time [19], [20], this new source of information opens up exciting perspectives not only for understanding past outbreaks, but also for unraveling the transmission routes of ongoing outbreaks and subsequently adapting public health responses.

Integrated analysis of both epidemiological and sequence data clearly would maximize our ability to reconstruct transmission trees, but there are methodological and computational challenges. These challenges center on constructing and evaluating a unified likelihood for both the genetic and epidemiological data. One of the first attempts at integrated analysis [21] used phylogenetic trees to constrain the set of transmission trees then explored by an epidemiological transmission tree inference algorithm. An alternative approach [22] highlighted limitations of phylogenetic methods for reconstructing densely sampled outbreaks, and proposed an alternative graph theoretic approach for reconstructing ‘genetically parsimonious’ transmission trees, *i.e.* trees implying the smallest number of genetic changes amongst the sampled isolates. While simple and fast, this method also has a number of limitations: dates of infection are not inferred, the probability of a given transmission event cannot be assessed, and unobserved cases or multiple introductions of the disease cannot be detected. Substantive methodological developments have been made by Ypma *et al.* [23] and subsequently by Morelli *et al.* [24], both of which proposed unified likelihoods for genetic and epidemiological data to analyze livestock disease outbreaks (avian influenza H7N7 [23] and foot-and-mouth disease [24]). However, those methods require that the outbreak has a single introduction event and that all cases are observed, which limits their applicability to restricted epidemic contexts.

Here we introduce a novel and generic framework for the reconstruction of disease outbreaks based on pathogen genetic sequences and collection dates. We use the distribution of the generation time (*i.e.* time interval between a primary and a secondary infection) [7], [8] to define the epidemiological likelihood of a given transmission tree. This is coupled with a simple model of sequence evolution defining the probability of the genetic changes observed between the pathogen genomes along a chain of transmission. Our model is embedded within a Bayesian framework allowing estimation of dates of infections, mutation rates, separate introductions of the pathogen, the presence of unobserved cases, and the transmission tree. Estimate of the effective reproduction number over time, *R*(*t*), can also be obtained. As an improvement over previous approaches [23], [24], our method does not require all cases to be observed or there to be a single introduction event which triggers an outbreak. After evaluating the performance of our method using simulated outbreaks, we illustrate our approach by analyzing the 2003 Severe Acute Respiratory Syndrome (SARS) outbreak in Singapore [10], [11], [25]. Our method is implemented in the package ‘*outbreaker*’ for the R software [26] and represents the first widely available tool for the reconstruction and analysis of disease outbreaks from genomic data.

## Results

### General results on simulated data

We analysed simulated outbreaks to assess the performance of our method under a variety of conditions, including different basic reproduction numbers (*R*_0_), sampling coverage, rates of evolution, and generation time distributions, with our base scenario resembling an influenza-like illness (Table 1). The outbreak size varied from 10 to nearly 200 infections in a fixed population of 200 susceptible hosts (plus imported cases), with a median sample size of 110 (quartile range: [66–132], Fig. S1). Wherever applicable, reported results refer to the marginal distributions.

**Table 1:** Parameters of the simulated outbreaks.

Parameter	Possible values	Label
Basic reproduction number (R^0^)	1.1	Low R
Basic reproduction number (R_0_)	**1.5**	Base
Basic reproduction number (R_0_)	4	High R
Generation time distribution	short (1.5, 1, 4)*	Short generation
Generation time distribution	average (2, 0.7, 5)*	Base
Generation time distribution	long (6, 3, 20)*	Long generation
Mutation rate**	0	No mutation
Mutation rate**	**1×10^−4^**	Base
Mutation rate**	2×10^−4^	Fast evolution
Genome length	**10,000**	[constant across simulations]
Rate of imported cases	0	No import
Rate of imported cases	**0.05**	Base
Rate of imported cases	0.2	Many imports
Proportion of cases sampled	0.25	75% missing cases
Proportion of cases sampled	0.50	50% missing cases
Proportion of cases sampled	0.75	25% missing cases
Proportion of cases sampled	**1**	Base

Values indicated in bold correspond to the base simulation. Every other value was changed individually from the base simulation, giving one unique simulation setting. For every setting, 50 independent simulated epidemics were obtained. The minimum outbreak size was set to 10 cases (smaller outbreaks were discarded). Labels are used throughout the text to identify unique simulation settings.

* the first two figures refer to the mean and standard deviation of the gamma distribution, before discretization; the third value is the date after which the distribution is truncated to zero.

** per site and per generation.

**Figure 1:**
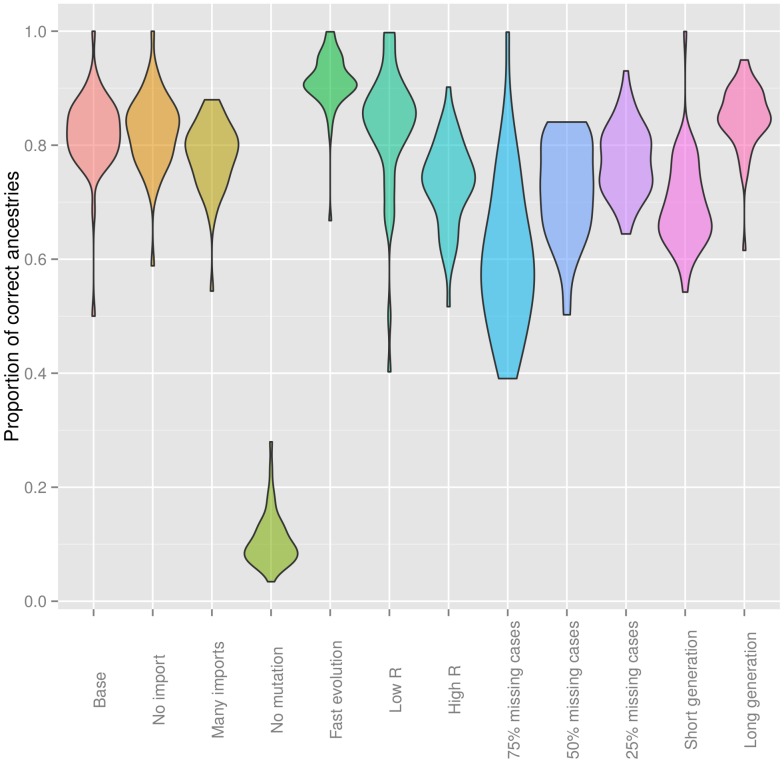
Quality of the transmission tree reconstruction in simulated datasets. This violinplot represents the proportion of correctly inferred transmissions in the consensus ancestries, obtained by retaining the most frequent infectors in the posterior trees for each case. Each colored ‘violin’ represents the density of points for a given simulation setting, indicated on the x-axis (see Table 1 for details).

Transmission trees were overall very well reconstructed, with 70% to 90% of true ancestries being recovered in most simulation settings (Fig. 1 and Table S1 in Text S1). Better results were achieved when the sampling coverage was high (compare settings ‘Base’ to 75%, 50% and 25% of missing cases). In the absence of genetic information, the transmission tree was very difficult to infer (setting ‘No mutation’). Differences in basic reproduction numbers (settings ‘Low R’ and ‘High R’) and in the shape of the generation time distribution (settings ‘Short generation’ and ‘Long generation’) induced some variation in the proportions of successfully recovered ancestries, although these remained satisfying in every case (Fig. 1 and Table S1 in Text S1). Dates of infections were inferred with accuracy in most settings (Fig. S2 and Table S1 in Text S1). However, this result was mostly driven by the shape of the generation time distribution, with broader distributions leading to greater uncertainty in the dates of infection (Fig. S2). While perfectly inferred in fully sampled outbreaks, the number of generations between ancestor and descendents became ambiguous as the proportion of missing cases increases (Table S1 in Text S1). Mutation rates were also mostly well estimated (Table S1 in Text S1, Fig. S3), albeit with a tendency to over-estimation. This bias was stronger when sampling grew sparser (settings with 75% and 50% missing cases), and to a lesser extent when the number of imported cases grew large (setting ‘Many imports’). Detailed investigation of individual simulations suggested that misdetection of imported cases and increased numbers of erroneous ancestries may be responsible for over-estimating the mutation rates in these settings. The inference of sampling coverage varied largely amongst different simulation settings (Table S1 in Text S1, Fig. S4): well recovered in fully sampled outbreaks, it was largely overestimated in sparse samples (settings with 75%, 50% and 25% missing cases), and slightly underestimated with longer generation time.

**Figure 2:**
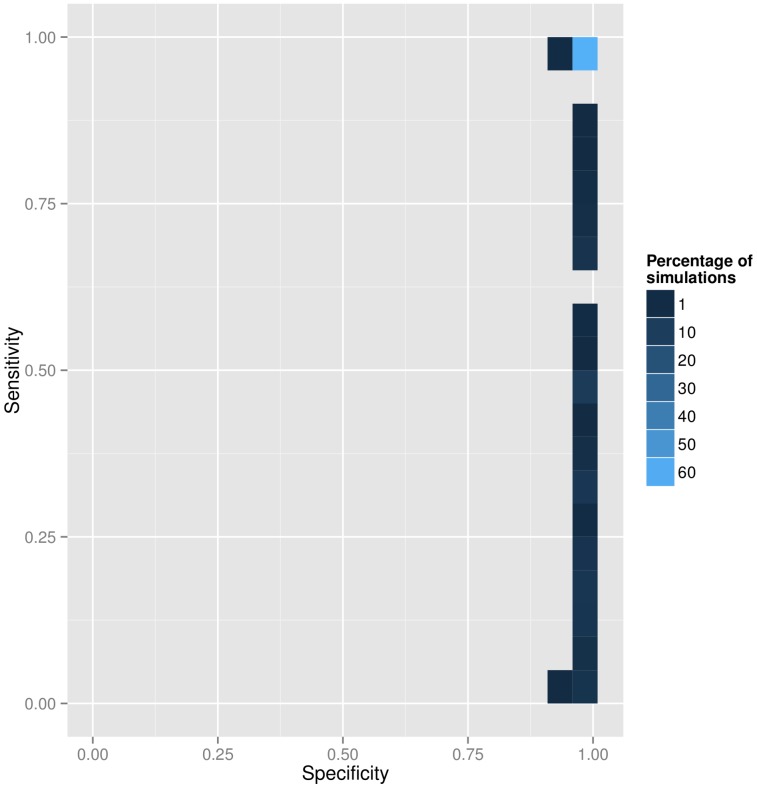
Detection of imported cases. This figure shows the specificity and sensitivity of the procedure for detecting imported cases based on the identification of genetic outliers. Colored rectangles represent the percentage of simulations within a given specificity/sensitivity range. All simulation settings were pooled for this analysis.

The detection of imported cases showed excellent specificity and good sensitivity pooling results across the simulated datasets examined, with a majority of simulations exhibiting perfect results (Fig. 2). However, substantial variations were observed between simulation settings (Fig. S5, Table S1 in Text S1). Unsurprisingly, detection of imported cases was more difficult when imported cases were more frequent and when a higher fraction of cases was unobserved. With longer generation times, the larger numbers of mutations accumulated between ancestors and descendents made the detection of genetic outliers, and thus of imported cases, nearly impossible (Fig. S2).

### Inferring effective reproduction numbers

**Figure 3:**
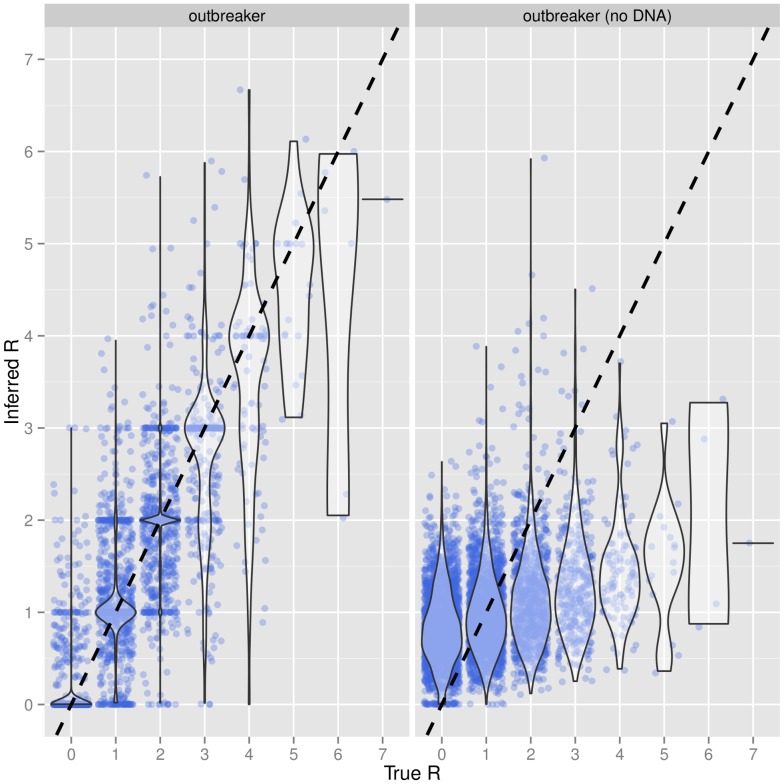
Inference of individual effective reproduction numbers. This violinplot shows the estimates of individual effective reproduction numbers (*R*) for simulated outbreaks with the ‘Base’ setting (see Table 1), based on 50 simulated epidemics, with (left) or without (right) using genetic information in the model. Each dot represents an infected individual. The dashed line indicates identity.

While our model does not explicitly estimate the effective reproduction number ‘*R*’ (i.e., the number of secondary cases per infected individual), this quantity can easily be computed from the posterior trees. Our ‘base’ simulations show that reliable estimates of *R* at an individual level can be obtained when genetic information is available (Fig. 3, left). In contrast, such inference was impossible in the absence of genetic data (Fig. 3, right).

To gain a better understanding of disease outbreak dynamics, identifying systematic heterogeneity in *R* across cases is also essential. To assess whether our approach could detect such heterogeneity, we implemented two types of simulations in which there were systematic differences in infectivity between groups of hosts. In a first set of simulations, the host population was divided into two groups of equal sizes (*e.g.* adults and children) with low and high infectivity (infectivity in one group was twice that of the other group, with equal susceptibility). In the second setting, we included rare (5%) super-spreaders, who had the same susceptibility to infections as non super-spreaders, but were 13-fold more infectious. In both sets of simulations, infectivity was fixed for each individual at the beginning of the simulations. The classification of individuals into super-spreaders and regular spreaders was considered as known when comparing estimated reproduction numbers.

**Figure 4:**
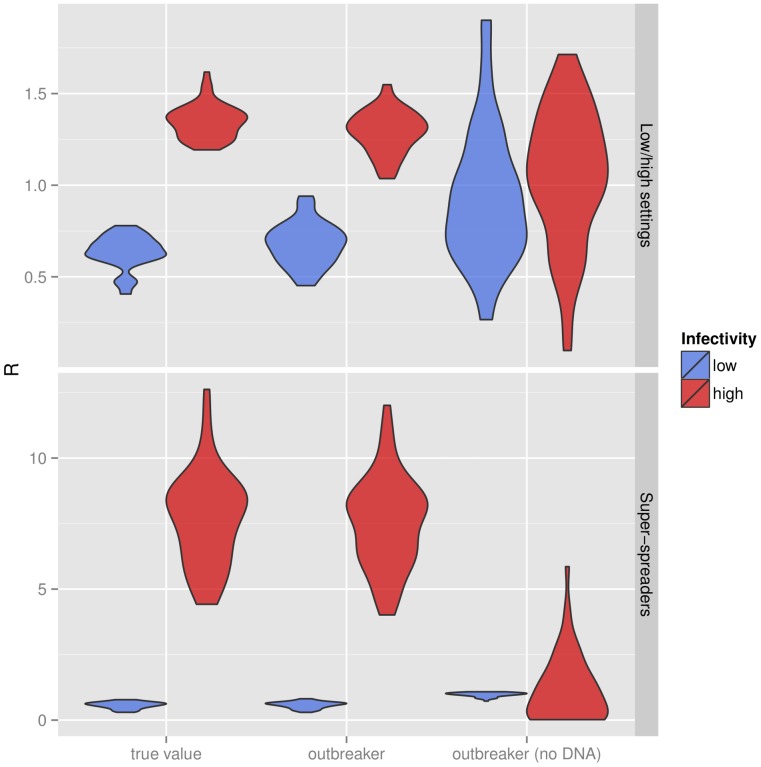
Detection of group-level heterogeneity in infectivity. This violinplot shows actual and estimated values of effective reproduction numbers (*R*) at an individual level, for outbreaks simulated with two groups of hosts having contrasted infectivity (‘Low’ and ‘high’). The top panel corresponds to simulations with equally-sized groups (‘Low/high settings’), while the bottom panel corresponds to simulations with super-spreaders.

Results showed that our method was able to recover contrasted infectivity between different groups (Fig. 4, S6, 7, 8, 9). In the simulations with equally-sized groups, the overall distributions of *R* for each group were almost perfectly recovered (Fig. 4, top panel), while values of *R* at an individual level were also well estimated (Fig. S6). Importantly, when ignoring the genetic information, differences between groups were barely detectable (Fig. 4 and S7). Similar results were observed in simulations including super-spreaders (Fig. 4, bottom panel), in which estimates of *R* values at an individual level were excellent when using genetic information (Fig. S8), and very poor without it (Fig. S9). The reconstruction of average *R* values over time was not improved by the inclusion of genetic information (Fig. S10, S11), which is unsurprising as this mainly depends on correctly inferring the dates of infections, which was unaffected by the absence of genetic data (Fig. S1, S2).

### Re-analysis of the 2003 SARS outbreak in Singapore

We analyzed data collected during the beginning of a SARS outbreak which took place in Singapore in 2003 [10], [25]. Previous studies proposed different reconstructions of this outbreak based on indirect contact tracing information and genetic data, and while all agreed on the necessity to combine these two streams of information, a clear consensus on the initial transmission tree has not been reached [10], [11], [25]. Here, we aimed to reconstruct the early stage of this outbreak using 13 full SARS genomes collected from the putative index patient and primary and secondary cases, and previously published estimates of the generation time distribution [27] (Fig. S12).

The genetic diversity amongst isolates was limited, with less than 15 mutations separating any pair of genomes (Fig. S13). For most cases, transmission events could not be readily inferred from the phylogenetic tree (Fig. S14). According to previous estimates of the mutation rate [25], we expect that most direct transmissions (>99%) will exhibit between 0 and 5 mutations. Using this result, we performed a simple graph analysis to derive possible clusters of direct transmissions, which suggested the existence of one main cluster of cases that may be linked directly, the remaining 4 isolates falling into three groups (Fig. S15). However, this crude analysis only relied on genetic diversity, and did not take into account information on the collection dates of the isolates or on the duration of the infectious period.

**Figure 5:**
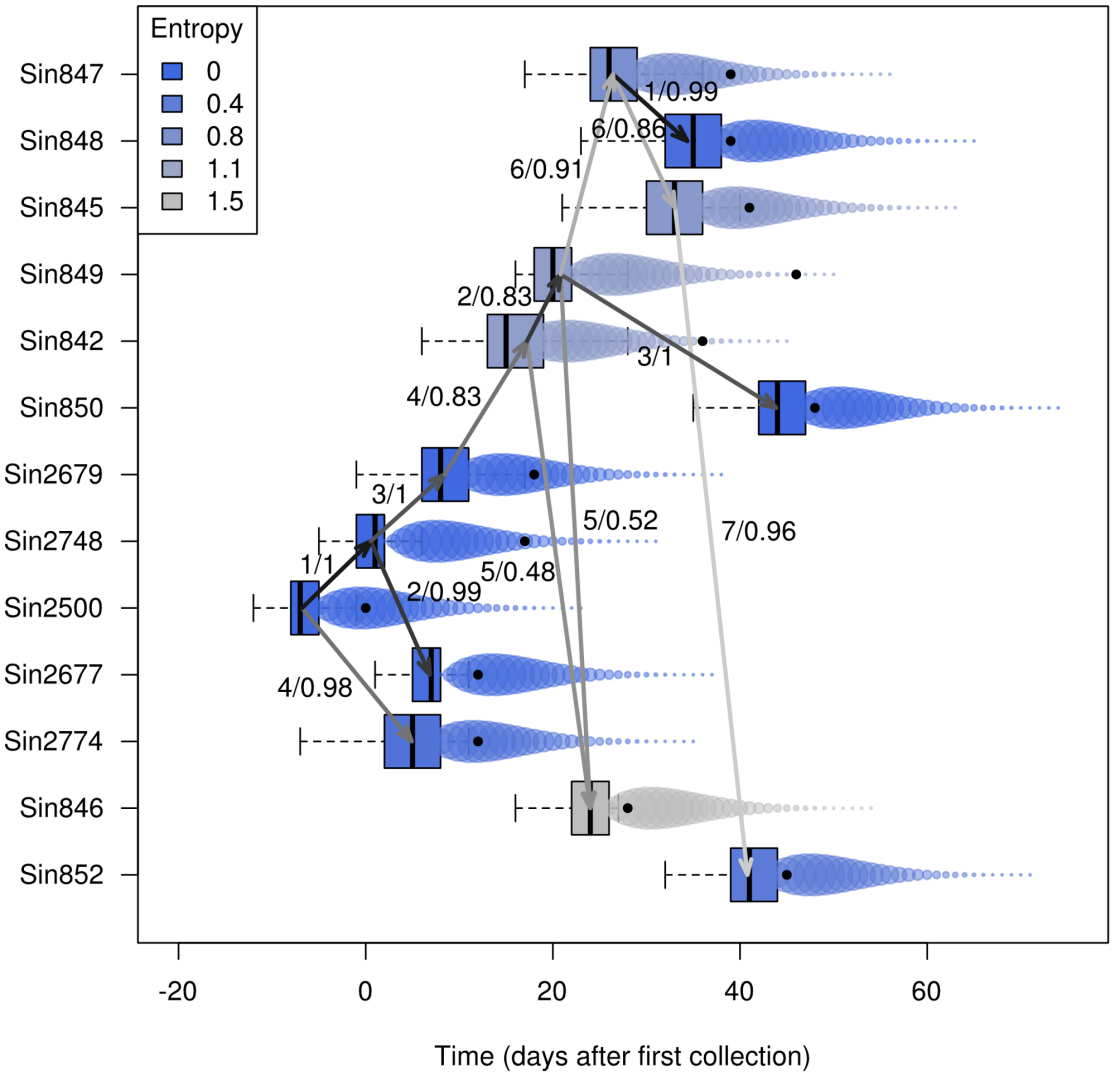
Results of the analysis of the SARS data using *outbreaker*. This figure summarizes the reconstruction of the outbreak, showing putative transmissions (arrows) amongst individuals (rows). Arrows represent ancestries with a least 5% of support in the posterior distributions, while boxes correspond to the posterior distributions of the infection dates. Arrows are annotated by number of mutations and posterior support of the ancestries, and colored by numbers of mutations, with lighter shades of grey for larger genetic distances. The actual sequence collection dates are plotted as plain black dots. Bubbles are used to represent the generation time distribution, with larger disks used for greater infectivity. Shades of blue indicate the degree of certainty for inferring the origin of different cases, as measured by the entropy of ancestries (see methods and equation 12): blue represents conclusive identification of the ancestor of the case (low entropy), while grey shades are uncertain (high entropy).

**Figure 6:**
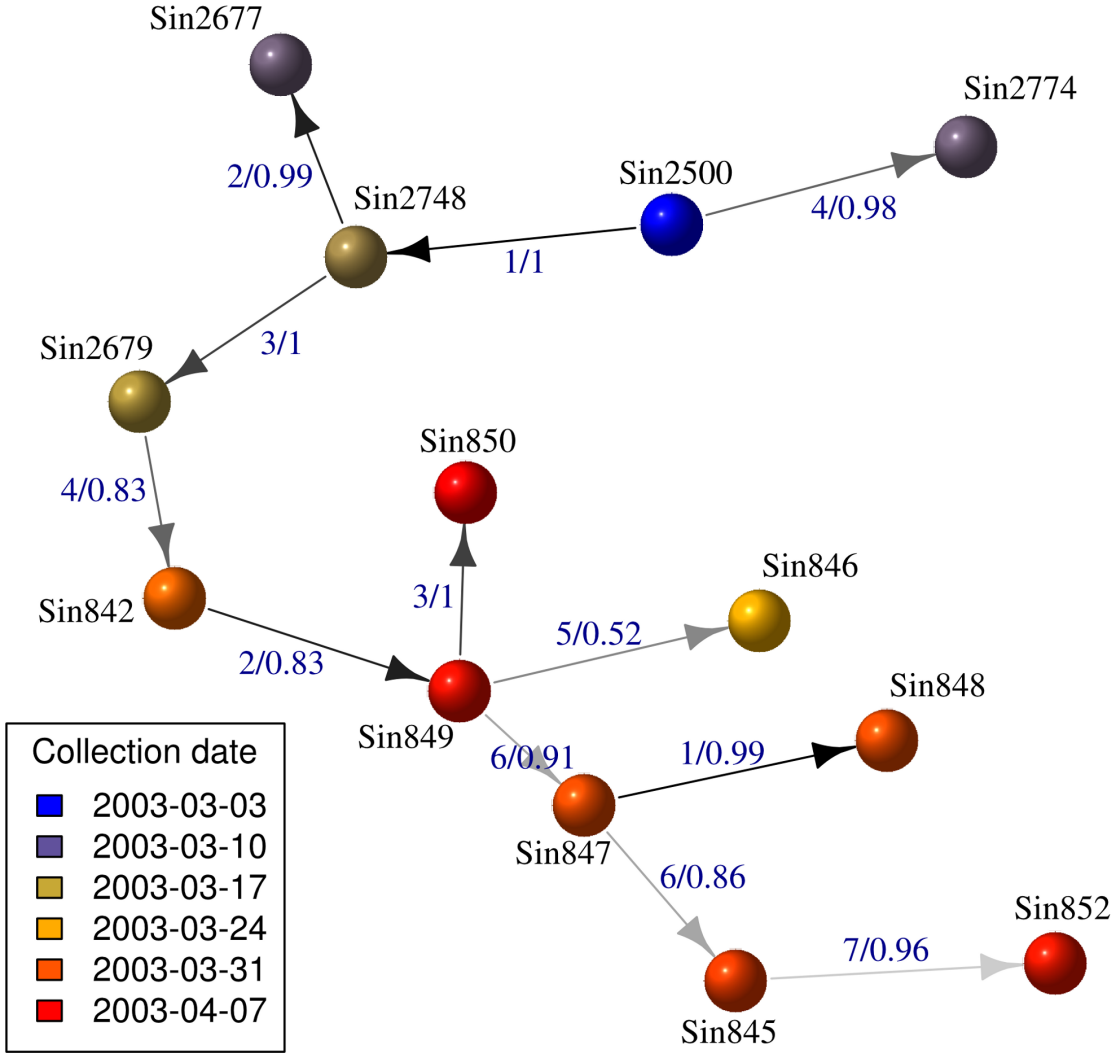
Consensus transmission tree reconstruction of the SARS outbreak. This figure indicates the most supported transmission tree reconstructed by *outbreaker*. Cases are represented by spheres colored according to their collection dates. Edges are colored according to the corresponding numbers of mutations, with lighter shades of grey for larger numbers. Edge annotations indicate numbers of mutations and frequencies of the ancestries in the posterior samples.

We used *outbreaker* to exploit all these data simultaneously. Results of the inferred likely scenarios (Fig. 5 and 6) show that for half of the cases, a well-supported ancestor can be identified from the data (see also Fig. S16). These correspond to all of the first and second generations of infections (Sin2677, Sin2679, Sin2748, Sin2774) and to the last sampled case (Sin850). Ancestries of most cases were compatible with a single generation, although one or two unobserved infections may have taken place between Sin849 and Sin850 (Fig. S17). We found no evidence for separate index cases after Sin2500, in agreement with contact tracing information [10], [11], [25]. However, the small number of cases may impair the detection of outliers and thus the identification of imported cases, so that multiple introductions of the pathogen cannot be ruled out.

The most recent investigation of this outbreak suggested a dual introduction of the pathogen, with a separate index case (Sin2679) nearly 20 days after the initial index case Sin2500 [10], [11], [25]. This may be deemed surprising as this case is genetically close to some preceding cases (Fig. S14, S15). Here, our results suggest that Sin2679 would in fact be part of the second generation of infection, and was infected by Sin2748 (Fig. 5 and 6). Indeed, while the collection dates of Sin2748 and Sin2679 are relatively close, the generation time of SARS (Fig. S12) may have allowed for this transmission to occur. Closer examination of the patterns of mutations between Sin2500, Sin2748 and Sin2679 bring further support to this scenario (Fig. 6, Data S3). Indeed, the four mutations separating Sin2500 from Sin2679 are the simple addition of the mutations accumulated on the chain of transmission, from Sin2500 to Sin2748 (position 26,430: a→g), and from Sin2748 to Sin2679 (18,284: c→a; 19,086: t→c; 23,176: c→t).

## Discussion

Building on past work [23], [24], we have presented a flexible analytical framework for the reconstruction of densely sampled outbreaks from epidemiological and sequence data. We extended previous work by accounting for unobserved cases and proposing a new approach for identifying multiple introductions of the pathogens based on the detection of genetic outliers. Our method is also the first tool for outbreak reconstruction widely available as a free software (the R package ‘*outbreaker*’) and able to run on standard desktop computers. The analysis of simulated data suggests that our approach will be applicable to a wide range of pathogens with various basic reproduction numbers, generation time distributions, and genetic diversity. We have shown how our approach can be used to infer effective reproduction numbers at an individual level. Importantly, this allows for detecting differences in infectivity of different groups of cases, and for the identification of super-spreaders. Our results suggest that while epidemiological data may suffice for the estimation of mean aggregated quantities such as the mean effective reproduction number, *R*, genetic data are useful to tease individual heterogeneities apart.

As in other tree reconstruction methods [2], [7], [28], [29], we did not explicitly model the population of susceptible individuals. This is because information on individuals who were not infected during the outbreak (the “denominator” data) is quite often unavailable. Compared with case-only analyses, availability of denominator data also makes it possible to estimate the force of infection and risk factors for infection [4]. We note that our framework could easily be extended to model the uninfected population. This could be done by modifying our likelihood so that the probability of the time of infection of a case would be based on an explicit model of the force of infection; individuals not infected during the outbreak would also contribute to the epidemiological likelihood as is standard in such situations [4]. Integrating and validating these additional features in our approach will be the subject of future research.

Our method relies on several assumptions which can be used to define the scope of its possible applications. The most important element in this respect is the proportion of cases represented in the sampled data, and thus often the scale of the epidemics considered. Our approach aims to reconstruct ancestries in closely related cases. As such, it should be most useful for detailed outbreak investigations. While the reconstruction of transmission tree seems relatively robust to large proportions of unobserved cases (up to 75% of missing cases, Fig. 1), our method is clearly tailored to densely sampled outbreaks, and not meant for the analysis of large-scale, more sparsely sampled epidemics. In such cases, phylogenetic methods are preferred as they explicitly reconstruct unobserved common ancestors of the sampled pathogen genomes, and can be used to infer, if not the transmission tree, the past dynamics of the disease [30], [31], [32].

One of the novelties of our approach is the detection of imported cases, which are identified as genetic outliers. While this method should be useful to detect separate introductions of different pathogenic lineages in an epidemic, it may be sensitive to other events prone to creating genetic outliers, such as sequencing errors or recombination. Care should therefore be devoted to ensuring data quality and filtering out polymorphism due to recombination. Moreover, the assumption that imported cases are genetically distinguishable from other cases may not always be true, especially when multiple introductions take place from a closely related lineage. Such cases cannot be detected by genetic data only, and would require other sources of information (e.g. contact tracing) to be considered. In this respect, an interesting feature of *outbreaker* is the ability to fix known imported cases (as well as any other known transmissions) before reconstructing the transmission tree.

Another important point is that following a previous, widely-used approach for the analysis of outbreaks [7], we assume the distributions of the generation time and of the time from infection to sample collection to be known. In some situations such as outbreaks of new emerging pathogens, accurate estimates of the generation time may not be readily available. In this case, a conservative approach should allow for a wide range of possible times to infection, at the expense of increased uncertainty in the inferred ancestries. As our method is numerically efficient for the analysis of small outbreaks, we suggest testing different generation time distributions to assess the robustness of the results. As a longer-term alternative, our approach could be extended to include an explicit parameterization and estimation of the generation time distribution.

More fundamentally, the use of a generation time distribution also implies that our method is less appropriate for diseases in which long periods of asymptomatic carriage are frequent. For instance, bacteria such as *Staphylococcus aureus* can cause infections after months of asymptomatic colonization of the host, but may equally cause outbreaks of cases linked by only a few days [12], [33]. In such cases, the collection dates of isolates effectively carry less information about possible transmissions, which would hamper our current approach. However, our model could be adapted to the analysis of carried pathogens by incorporating specific data on known exposures (e.g. shared occupancy on a hospital ward) [34], [35], [36].

Moreover, carried pathogens are also more likely to cause multiple colonizations of the host, resulting in several lineages coexisting within the same patient. Our model assumes that a single pathogen genome exists within each host, and is therefore not designed to account for multiple infections. A simple workaround would consist in duplicating cases of multiple infections into single infections, assuming that multiple infections are made of independent, single colonization events. However, this would not allow for disentangling multiple infections from mere within-host evolution of a single lineage. A more satisfying approach would consist in modeling explicitly the evolution of isolates within host, but this will likely result in a much more complex model and is beyond the remit of our current approach.

A major simplification made in our model, that could be relaxed in future work, is that we do not consider within host diversity of pathogens. Within-host diversity is particularly prominent in pathogens that infect a host for a long time relative to their within-host replication cycle (e.g. HIV or Hepatitis C Virus), pathogens that can be carried for a long time (e.g. *Staphylococcus aureus*), pathogens where the infectious inoculum is large (e.g. blood-transmitted HIV), or super-infection is frequent (e.g. *Streptococcus pneumoniae* in hyper-endemic settings). Limited host diversity leads us to assume that genomes sampled from infectors are effectively ancestral to genomes sampled from secondary cases, allowing us to equate phylogenetic and transmission trees. This substantially reduces the complexity of the inferential problem, and reduces by orders of magnitude the dimensionality of the space of linked augmented variables to be explored. The assumption of no within-host diversity will likely be appropriate for acute infectious pathogens in outbreaks, but will also be relatively appropriate for situations where there is a strong bottleneck on diversity upon transmission and limited opportunities for superinfection, such as sexually transmitted HIV. Inclusion of within-host diversity in the model inference is an important but likely complex task, though efficient approximations may be possible. A related development will be the inclusion of multiple samples per individual, used to sample cross-sectional and longitudinal genetic diversity within infected hosts. Another somewhat simpler extension would be the inclusion of a ‘relaxed’ molecular clock, which would allow accounting for heterogeneities in mutation rates amongst different pathogen lineages.

Finally, we wish to emphasize the importance of including all available prior information in the analysis. Because the estimates of parameters governing an outbreak are often correlated, accurate knowledge of one can be used to refine the estimation of the others. For instance, specifying known transmission chains or imported cases will improve the estimation of the mutation rates, as well as the overall reconstruction of the transmission tree. Conversely, fixing the mutation rate to its ‘true’ value (or a good estimate thereof) is likely to improve the detection of imported cases. As currently implemented, our method allows for fixing any parameter as well as individual ancestries, which are used in the likelihood computations but not changed during the MCMC. This feature should be especially useful for incorporating known transmission events or introductions of the pathogen into the population, based for instance on clinical investigations and contact tracing information. However, results of contact tracing studies should always be considered cautiously, and could be contradicted by the analysis of corresponding sequences, as illustrated by the SARS outbreak in Singapore.

There are other promising avenues for incorporating various streams of information into our approach. The likelihood of our model allows for additional ‘plug-in’ terms for individual transmissions, which could be used to model spatial dispersion processes as well as movement over a contact network. Therefore, we hope that the present method will not only be applied widely, but also motivate further developments for the investigation of infectious disease outbreaks.

## Methods

### Model of disease transmission

#### Model notations

We developed a discrete-time stochastic model for reconstructing likely transmission trees of an outbreak based on pathogen genetic sequences and their collection dates (see notations summary in Table 2 and Figure S18). Our model considers a single pathogen genome for each case. We note 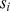 the genetic sequence of case 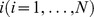, sampled at time 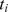. The function 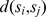 computes the number of mutations between 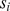 and 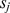, while 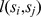 computes the number of nucleotide positions which can be compared between the two sequences. 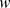 is the distribution of the generation time, defined as the time interval between the infection of an individual and his seeding of new secondary cases. 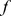 is the distribution of the time interval between infection and collection of an isolate. Both 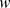 and 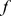 are assumed to be known, and are not part of the estimated parameters. Augmented data are used to model the transmission process, which is not observed directly [5], [34]. We denote 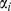 the index of the most recent sampled ancestor of case 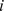, and 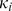 the number of generations separating cases 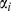 and 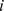 (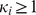). For imported cases, 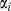 is fixed to 0. The date of infection for case 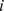 is denoted 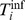. We use the simplest model of sequence evolution considering one single mutation rate (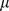), measured per site and per generation of infection. Unlike approaches based on strict molecular clocks (e.g. [24]), a generational clock models the accumulation of genetic diversity with new infections while overlooking within-host evolution [23]. Lastly, the parameter 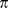 is the proportion of cases of the outbreak that have been sampled over the time span of the dataset, assuming a constant reporting rate over time.

**Table 2:** Notations used.

Symbol	Type	Description
*_i_*	Index	index of cases
*_N_*	Data	number of cases in the sample
*s_i_*	Data	sequence of case *i*
*t_i_*	Data	collection date of *s_i_*
*w*	Function	generation time distribution
*f*	Function	time-to-collection distribution
*d*(*s_i_*, *s_j_*)	Function	number of mutations between *s_i_* and *s_j_*
*l*(*s_i_*, *s_j_*)	Function	number of comparable nucleotides between *s_i_* and *s_j_*
*α_i_*	Augmented data	index of the most recent sampled ancestor of case *i*
*κ_i_*	Augmented data	number of generations between *α_i_* and *i*
	Augmented data	date of the infection of *i*
*μ*	Parameter	mutation rate, per site and per generation of infection
*π*	Parameter	proportion of cases of the outbreak sampled

#### Posterior distribution and full likelihood

Our model is embedded within a Bayesian framework. We denote *Y* the observed data, *A* the augmented data, and 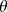 the model parameters. The joint posterior distribution of parameters and augmented data is defined as:
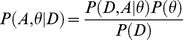
(1)which is proportional to:
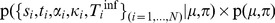
(2)where the first term is the likelihood of the data and augmented data, and the second, the joint prior distribution. Likelihood computations are described below. Priors and estimation procedures are described in Supporting Methods.

The likelihood is computed as a product of case-specific terms, in which we assume that all cases are independent conditional on their ancestries:
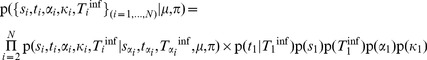
(3)where 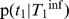 is the probability of the first collection date given the first infection date, and 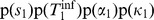 is a constant. In the case of partially sampled transmission chains, several cases could share some common (unsampled) ancestry, and would thereby no longer be independent conditional on their most recent sampled ancestor. It follows that in the general case, Eq. 2 is not a true likelihood but a composite likelihood [37] used to approximate the likelihood [24].

The general term of the pseudo-likelihood for case 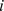 is:
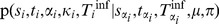
(4)which can be decomposed into:
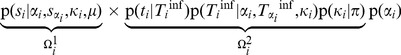
(5)where 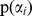 is a constant. We refer to 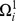 as the genetic pseudo-likelihood and to 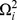 as the epidemiological pseudo-likelihood.

#### Genetic pseudo-likelihood

As in [22], mutations are modeled as features of the transmission events. This is a direct corollary of the assumption of no within host diversity. This approach has the advantage of being computationally very efficient, as only the genetic distances between isolates need to be known to compute the pseudo-likelihood of a transmission event, and all transmission events are independent. The genetic pseudo-likelihood of case 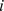 is defined as the probability of observing the genetic differences between the sequence 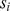 and the ancestral sequence 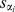 with 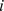 and 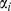 being separated by 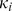 generations. In practice, if case 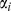 has not been sequenced, we look for another ancestral sequence by moving up the transmission chain, replacing 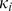 by the number of generations between the two compared sequences. Given the short timescale considered between pairs of sequences, reverse mutations are considered negligible. Accordingly, sites under strong selection such as immune epitopes or drug-resistance associated SNPs should be removed from the analyzed sequences. Assuming that all sites mutate independently and in the absence of reverse mutations, the genetic pseudo-likelihood 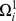 is given by:
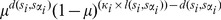
(6)

#### Epidemiological pseudo-likelihood

The epidemiological pseudo-likelihood 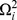 is computed as:
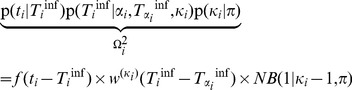
(7)The first term corresponds to the pseudo-likelihood of the collection date. The second term is the probability of the infection date for 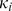 generations between the infection dates considered. 
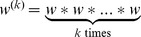
, where 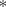 is the convolution operator. The last term is the probability of unobserved intermediate cases, modeled with a negative binomial distribution 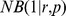 (equivalent to a geometric distribution with parameter *p*), indicating the probability of obtaining one ‘success’ (here, sampling a case) after 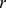 ‘failures’ (unobserved cases) with a probability of success 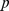.

#### Detection of imported cases

Imported cases are not explicitly included in the model, but detected using a preliminary run of the model, during which genetic outliers are identified and the corresponding cases classified as imported. The ancestry of these cases is fixed as ‘unknown’ in the second and final run. We use a leave-one-out procedure for detecting cases with outlying genetic log-likelihood which has been used previously in a similar context [38]. This approach defines the global influence 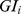 of case 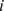 (considering genetic data only) as:
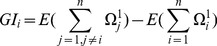
(8)where 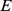 denotes the expectation of the corresponding terms, approximated by the average over a number of samples (50 by default) from the MCMC of the preliminary run. Large values of 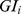 reflect unlikely numbers of mutations, and therefore a probable genetic outlier. By default, cases with a global influence greater than 5 times the average global influence are classified as outliers. While this threshold is arbitrary, it was determined empirically to have excellent specificity and appreciable sensitivity on a range of simulation settings (see Fig. 2).

#### Re-estimation of the mutation rate

Because our model uses a mutation rate expressed per generation of infection, estimated values cannot be readily compared to classical rates of evolution, typically expressed per unit of time. As a workaround, we can re-estimate a classical mutation rate from the distribution of posterior trees. The mutation rate can be inferred from one transmission event as the ratio of the number of mutations from ancestor to descendent and the amount of time separating the infection dates of these cases. For each tree, we compute the average mutation rate across all ancestries, which provides one estimate of the mutation rate for each posterior sample. This procedure is implemented in the function *get.mu* in *outbreaker*.

#### Implementation

Our approach is implemented in the R package *outbreaker* (version 1.1-0), freely available at: http://cran.r-project.org/web/packages/outbreaker/index.html.

### Simulation of disease outbreaks

#### Model

Outbreaks were simulated using the function *simOutbreak* in the package *outbreaker*. Each simulation starts with a single infection in a population of 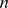 susceptible hosts. For simplicity, the same function was used for 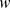 and 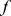. 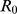 is the fixed basic reproduction number, and 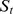 the number of susceptible hosts at time 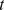. The probability for a susceptible individual to become infected on day *t* is:
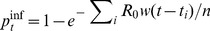
(9)At each time step, the number of new cases is drawn from a binomial distribution with 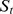 draws and a probability 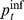. Infectors of a case infected at time *t_i_* are sampled from a multinomial distribution with probabilities:
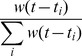
(10)In addition to endogenous cases, external cases are imported at a constant rate.

Mutations are simulated using a single mutation rate, all sites mutating independently. Pathogens of separate introductions of the disease (including the index case) are assumed to all coalesce to the same common ancestor ten generations ago.

#### Simulated scenarios

We evaluated the overall performance of the method using a basic scenario, and assessed the impact of different factors on the results by changing one aspect of the simulation at a time. These factors included the shape of the generation time distribution (from peaked to flat), the basic reproduction number (from 1.1 to 4), the mutation rates (from 0 to 2 mutations on average per generation and genome), the proportion of cases observed (from 0.25 to 1), the rate at which external cases are imported (from 0 to 0.2), and the proportion of sampled cases with DNA sequences (from 0.25 to 1). The different values for each element are summarized in Table 2. For each setting, 50 epidemics were simulated with 200 susceptible hosts and a minimum of 10 cases, and analyzed using *outbreaker* with the default settings, described in supporting information.

In addition, two other types of simulation were used to test our approach's ability to detect heterogeneous infectivity amongst cases. First, we generated outbreaks where the host population was divided into two groups of equal sizes, one being twice as infectious (equivalent *R*_0_ = 3) as the other (equivalent *R*_0_ = 1.5). Second, we simulated outbreaks with super-spreader dynamics, were 5% cases were super-spreaders, with an equivalent *R*_0_ of 20, while the rest of the population had an equivalent *R*_0_ of 1.5. In both cases, 50 outbreaks with minimum sizes of 10 cases were simulated using a single pathogen introduction and 100 susceptible hosts, and fully sampled outbreaks were analysed using *outbreaker*, fixing 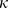 values to 1 generation and using defaults otherwise. For the super-spreader simulations, super-spreaders were identified first from the data and their reproduction number compared to that of the non super-spreaders. For such comparisons, effective reproduction numbers of the different groups were calculated based on cases during the whole outbreak.

### Analysis of the 2003 SARS outbreak in Singapore

Thirteen previously published full SARS genomes [10], [25] (Data S1) were obtained from Genbank and aligned using MUSCLE [39]. The resulting alignment contained 29,731 columns, 39 of which were polymorphic (Data S2). We used a generation time distribution modeled as a discretized gamma distribution with a mean of 8.4 days and a standard deviation of 3.8 days [27], using the function *DiscrSI* from the R package *EpiEstim* [29]. The same distribution was used for the the time to collection. Details of the parameters used to run *outbreaker* are provided in Supporting Methods. The statistical confidence in determining the ancestry of a given case was quantified using the entropy of the frequencies of the posterior ancestors. With 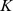 different ancestors of posterior frequencies 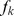 (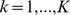), the entropy is defined as:
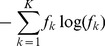
(11)The entropy is 0 if one of the 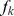, is 1, indicating high confidence in allocation of an ancestry, while larger values of the entropy indicate poorer confidence.

## Supporting Information

Data S1 **SARS genome data.** Information about the 13 SARS genomes collected from Genbank. The first column contains identifiers of the cases, while the second column contains Genbank accession numbers.
(CSV)

Data S2 **SARS genome alignment.** DNA alignment in fasta format of 13 SARS genomes collected during an outbreak in Singapore in 2003. Sequence labels contain the identifier of the case, and the collection date in format dd/mm/yyyy.
(FASTA)

Data S3 **List of mutations between pairs of SARS genomes.** This text file reports the output of the function ‘find*Mutations*’, implemented in the R package *adegenet*. The list of mutations from one genome to another is provided for all pairs of genomes in the SARS data. Genome labels match those of the fasta file provided as Data S1.
(TXT)

Figure S1 **Sample sizes of simulated datasets.** This violinplot represents the number of cases analysed in the different simulation settings. Symbols represent the densities of points across 50 independent replicates. Colors indicate different simulation settings (see Table 1 in main text for details).
(TIF)

Figure S2 **Inference of dates of infections in simulated datasets.** This violinplot represents the mean error in the inferred date of infection, in number of days from the true date. Symbols represent the densities of points across 50 independent replicates. These results are based on the posterior distributions of the infection dates. Colors indicate different simulation settings (see Table 1 in main text for details).
(TIF)

Figure S3 **Inference of the mutation rate in simulated datasets.** This violinplot represents the relative error in the inferred mutation rates. Mutation rates per unit of time were re-estimated from the posterior transmission trees using the function *get.mu* from the *outbreaker* package. Symbols represent the densities of points across 50 independent replicates. Colors indicate different simulation settings (see Table 1 in main text for details).
(TIF)

Figure S4 **Inference of the sampling coverage in simulated datasets.** This violinplot represents the mean error in the inferred sampling coverage (proportion of the outbreak sampled). Symbols represent the densities of points across 50 independent replicates. Colors indicate different simulation settings (see Table 1 in main text for details).
(TIF)

Figure S5 **Detection of imported cases in simulated datasets.** This violinplot represents the proportion of imported cases detected by the method. Symbols represent the densities of points across 50 independent replicates. Colors indicate different simulation settings (see Table 1 in main text for details).
(TIF)

Figure S6 **Inference of individual *R* with group-structured infectivity, using genetic information.** This violinplot shows the estimates of individual effective reproduction numbers (*R*) for outbreaks incorporating group-structured infectivity. Results are based on 50 replicates. Densities represent individuals from both groups, while colored symbols (circles, crosses) distinguish the groups. The dashed line indicates identity.
(TIF)

Figure S7 **Inference of individual *R* with group-structured infectivity, without genetic information.** This violinplot shows the estimates of individual effective reproduction numbers (*R*) for outbreaks incorporating group-structured infectivity. Results are based on 50 replicates, without the use of genetic information. Densities represent individuals from both groups, while colored symbols (circles, crosses) distinguish the groups. The dashed line indicates identity.
(TIF)

Figure S8 **Inference of individual *R* in presence of super-spreaders, using genetic information.** This violinplot shows the estimates of individual effective reproduction numbers (*R*) for outbreaks incorporating super-spreaders. Results are based on 50 replicates, without the use of genetic information. Densities represent all individuals, while colored symbols (circles, crosses) distinguish the super-spreaders from ‘normal’ individuals. The dashed line indicates identity.
(TIF)

Figure S9 **Inference of individual *R* in presence of super-spreaders, without genetic information.** This violinplot shows the estimates of individual effective reproduction numbers (*R*) for outbreaks incorporating super-spreaders. Results are based on 50 replicates. Densities represent all individuals, while colored symbols (circles, crosses) distinguish the super-spreaders from ‘normal’ individuals. The dashed line indicates identity.
(TIF)

Figure S10 **Example of reconstruction of the average effective reproduction number over time.** This figure illustrates the inference of *R* over time in one simulation (setting ‘base’) derived from posterior ancestries. The actual values of *R* are shown in red. Missing values correspond to time steps without new infections.
(TIF)

Figure S11 **Inference of the average effective reproduction number over time.** This violinplot shows the mean error (ME) in the estimated values of *R* over time, in basic simulated outbreaks (setting ‘base’), and in outbreaks incorporating group-structured infectivity (‘Low/high settings’) or super-spreaders (‘Super-spreaders’). Each box represents 50 independent replicates.
(TIF)

Figure S12 **Generation time distribution for SARS.** Probability mass function of the time between primary and secondary cases (*i.e.*, time after which a newly infected individual creates new infections).
(TIF)

Figure S13 **Distribution of pairwise genetic distances in the SARS data.** This histogram shows the distribution of the pairwise distances between the 13 SARS genomes of the 2003 Singapore outbreak, expressed in number of differing nucleotides.
(TIF)

Figure S14 **Phylogenetic tree of the SARS data.** Neighbor-Joining tree based on the Hamming distances (see Fig. S10) between the 13 SARS genomes of the 2003 Singapore outbreak. The tree is rooted to the most ancient isolate (Sin2500). Colors indicate time, with more ancient isolates in blue and more recent isolates in red. This tree was realized using the package *ape* for the R software.
(TIF)

Figure S15 **Graph connecting closely related genomes.** These clusters were defined using a graph approach where pairs of genomes are connected when they are distant by no more than 5 mutations from each other (function ‘*gengraph*’ from the R package *adegenet*). The resulting connected components form clusters represented using different colors. Numbers annotating the edges represent the number of mutations between pairs of genomes. For the sake of readability, the dates were removed from the labels of the sequences.
(TIF)

Figure S16 **Entropy of the ancestries inferred for the SARS data.** These entropies are computed from the frequencies of the different ancestries for each case. Low values indicate clear-cut ancestors for the corresponding case.
(TIF)

Figure S17 **Number of generation of the inferred ancestries in SARS data.** This barplot represents the posterior distribution of the number of generations in inferred ancestries for each case (rows).
(TIF)

Figure S18 **Outline of the transmission model.** This diagram illustrates the concepts and notations used in the transmission model, using a single transmission event. Data are represented in black, augmented data in blue, and parameters in red. For both time interval distributions (*w* and *f*), larger circles are used to indicate larger probabilities.
(TIF)

Figure S19 **Convergence of the MCMC for the analysis of SARS data.** This figure shows the posterior values of 6 independent MCMC (1,000,000 iterations each) used for the analysis of the SARS data. The burnin period chosen visually was 100,000 iterations.
(TIF)

Text S1 **Supporting methods and tables.** This file describes the priors and parameter estimation procedures used in *outbreaker*, as well as the settings used in the SARS outbreak analysis and the supporting table S1.
(PDF)
