# Supplementary figures and images for: Bayesian Reconstruction of Disease Outbreaks by Combining Epidemiologic and Genomic Data
Source: PLoS Comput Biol. 2014 Jan 23;10(1):e1003457. doi: 10.1371/journal.pcbi.1003457 (PMC3900386; doi:10.1371/journal.pcbi.1003457)

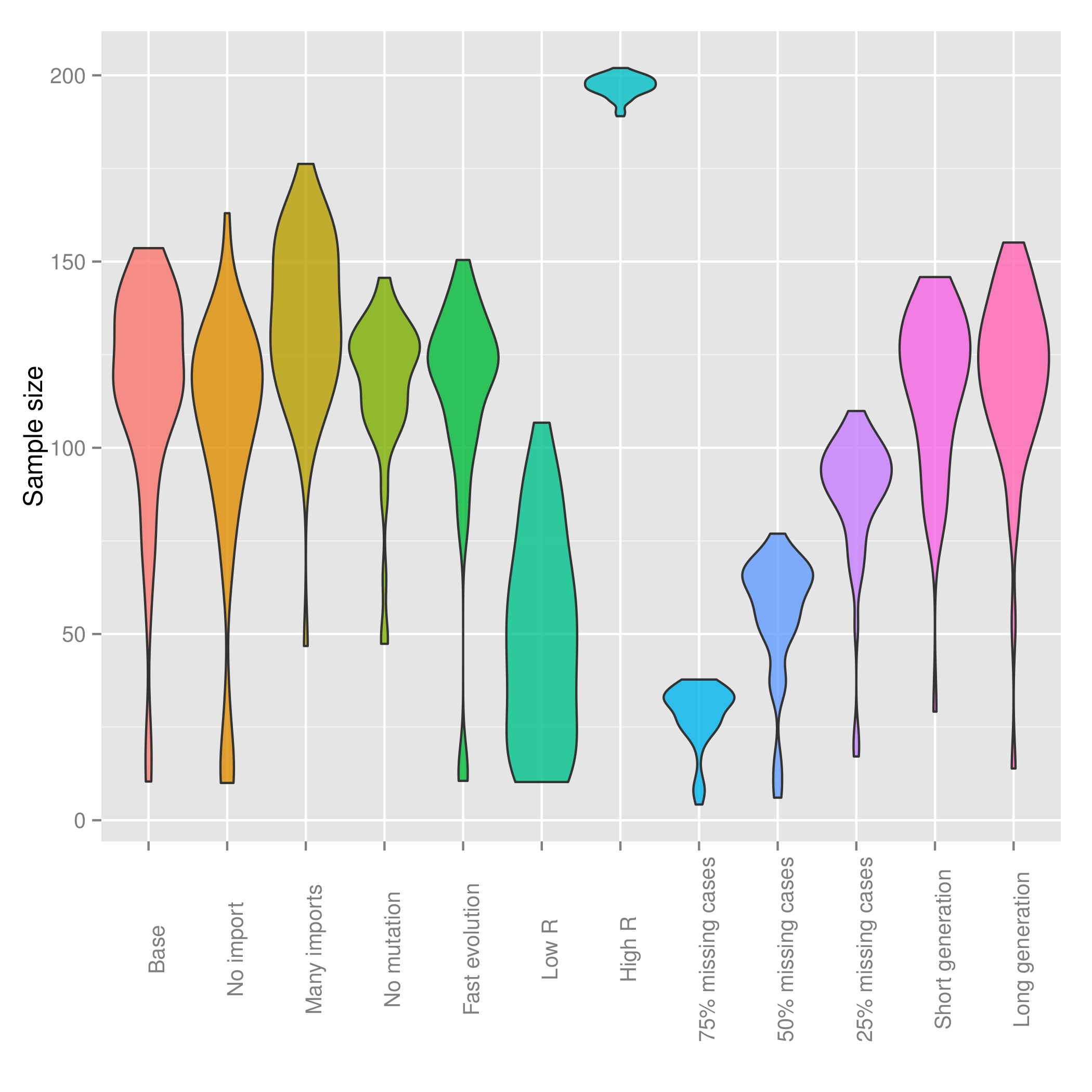

Supplement: Figure S1 — Sample sizes of simulated datasets. This violinplot represents the number of cases analysed in the different simulation settings. Symbols represent the densities of points across 50 independent replicates. Colors indicate different simulation settings (see Table 1 in main text for details). (TIF) [file pcbi.1003457.s004.tif]

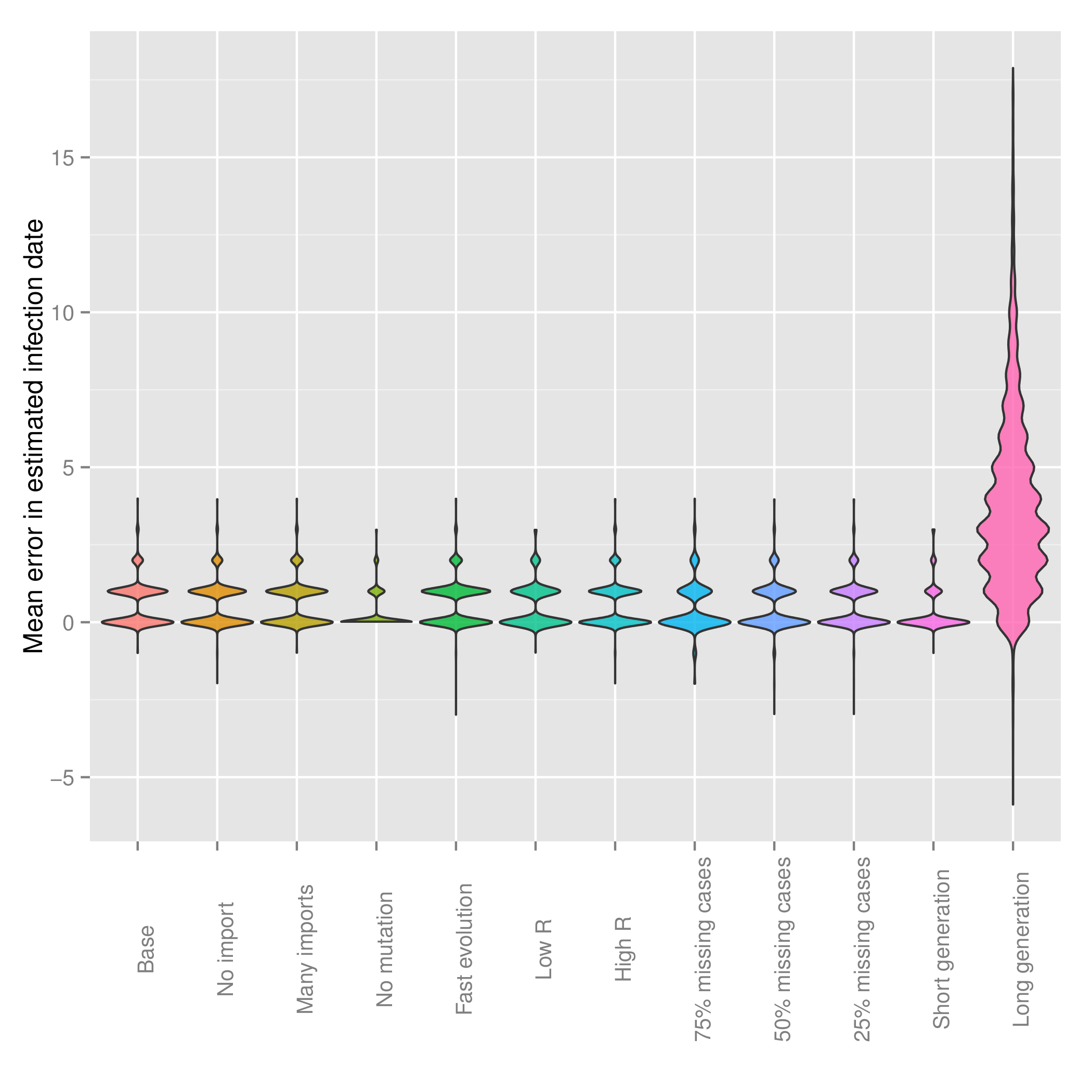

Supplement: Figure S2 — Inference of dates of infections in simulated datasets. This violinplot represents the mean error in the inferred date of infection, in number of days from the true date. Symbols represent the densities of points across 50 independent replicates. These results are based on the posterior distributions of the infection dates. Colors indicate different simulation settings (see Table 1 in main text for details). (TIF) [file pcbi.1003457.s005.tif]

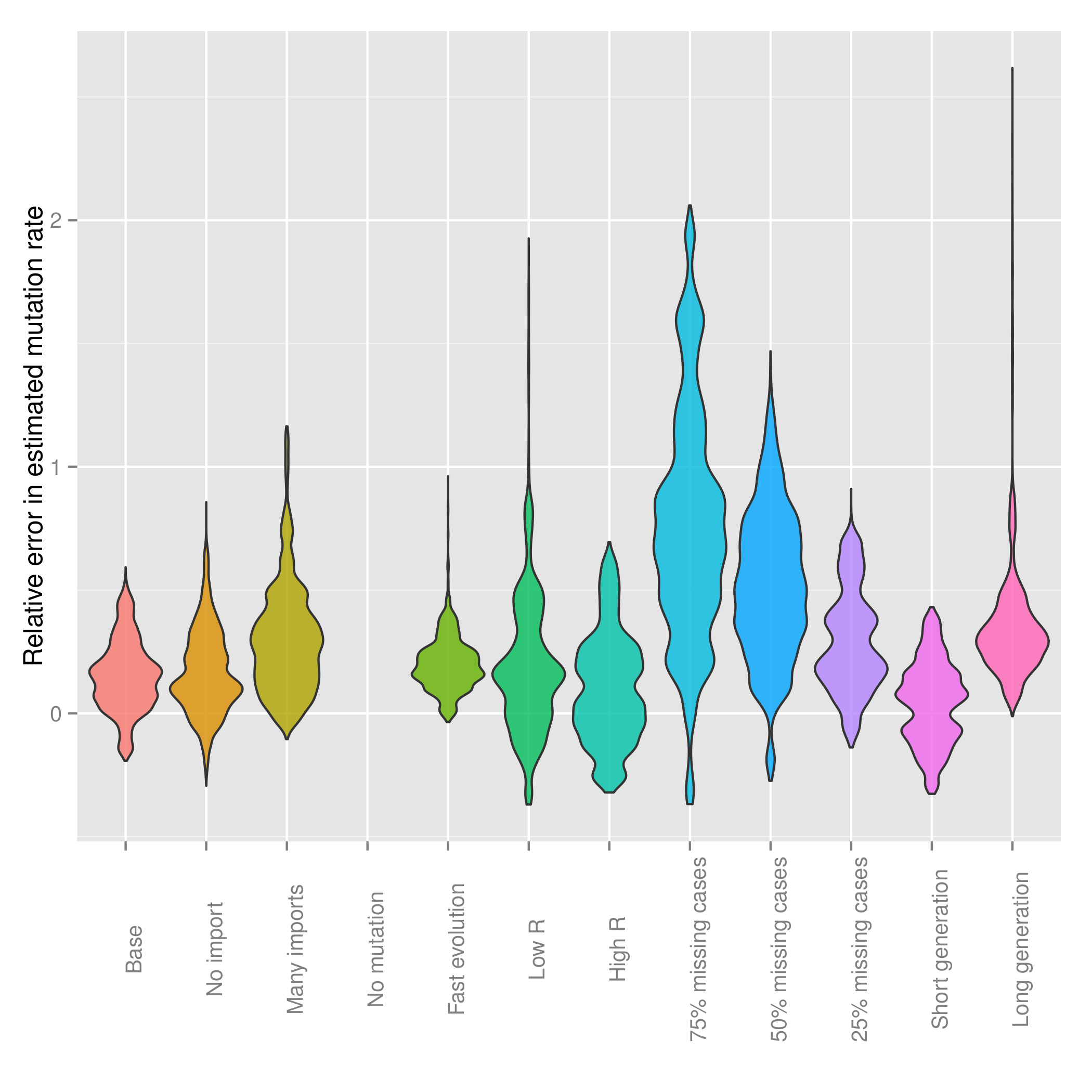

Supplement: Figure S3 — Inference of the mutation rate in simulated datasets. This violinplot represents the relative error in the inferred mutation rates. Mutation rates per unit of time were re-estimated from the posterior transmission trees using the function get.mu from the outbreaker package. Symbols represent the densities of points across 50 independent replicates. Colors indicate different simulation settings (see Table 1 in main text for details). (TIF) [file pcbi.1003457.s006.tif]

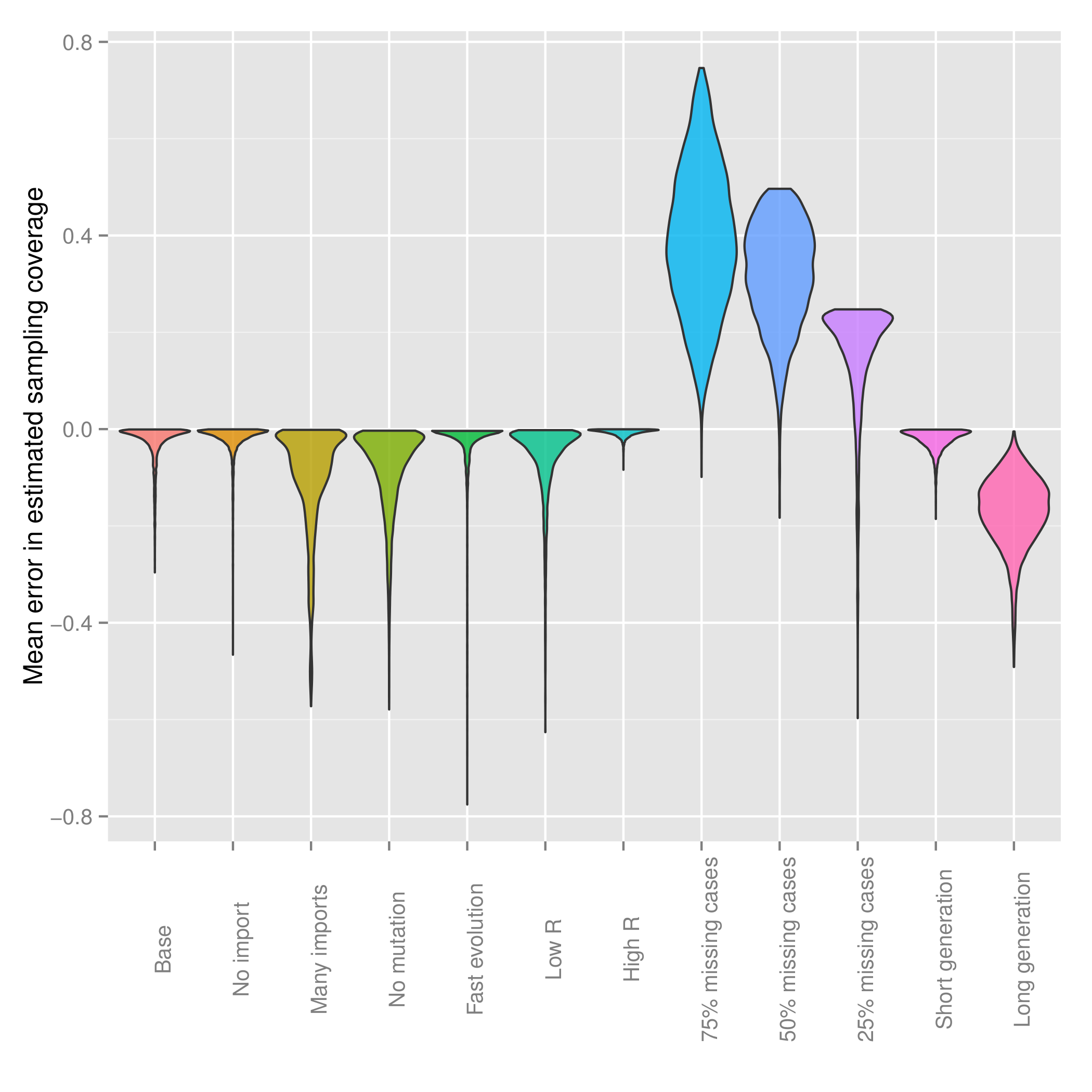

Supplement: Figure S4 — Inference of the sampling coverage in simulated datasets. This violinplot represents the mean error in the inferred sampling coverage (proportion of the outbreak sampled). Symbols represent the densities of points across 50 independent replicates. Colors indicate different simulation settings (see Table 1 in main text for details). (TIF) [file pcbi.1003457.s007.tif]

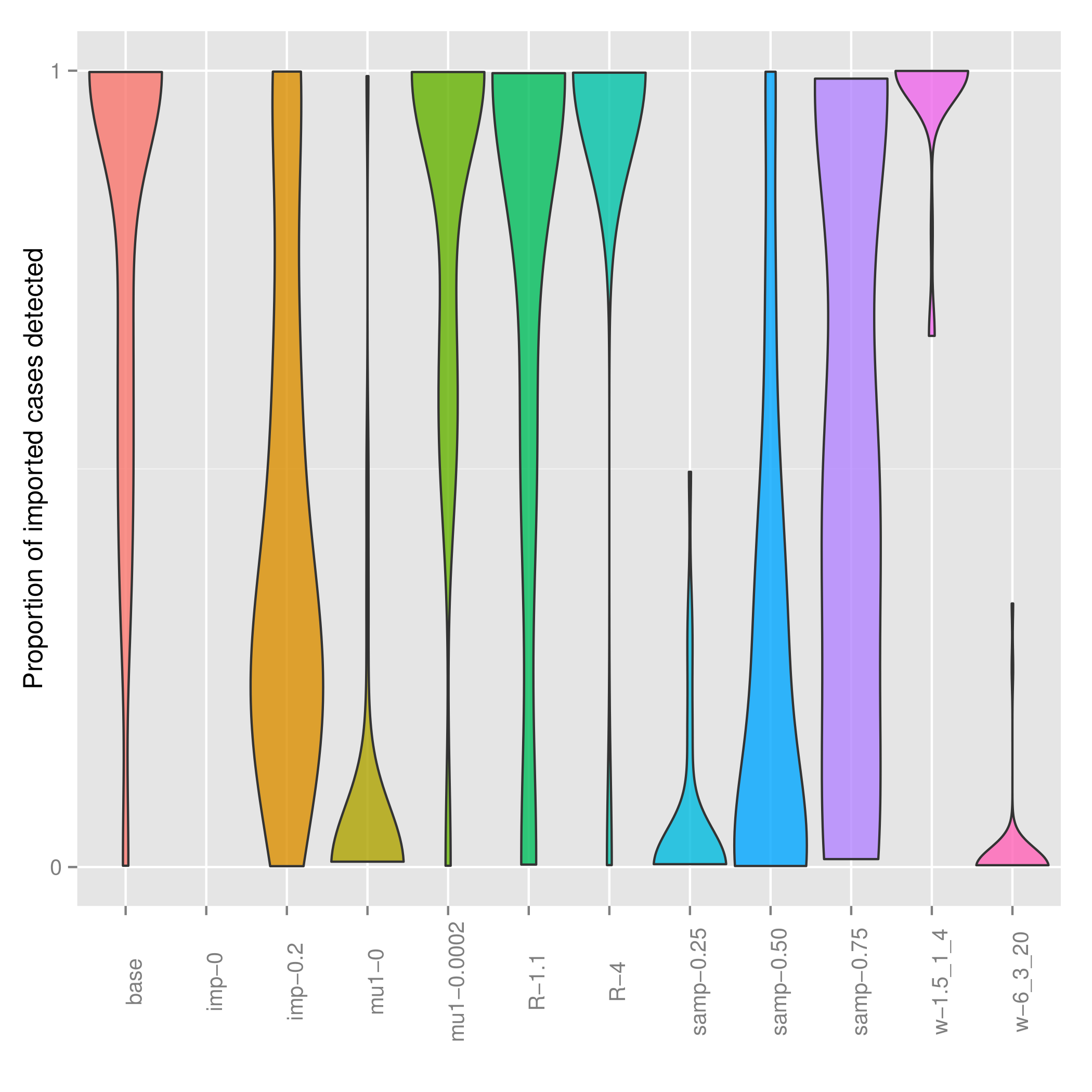

Supplement: Figure S5 — Detection of imported cases in simulated datasets. This violinplot represents the proportion of imported cases detected by the method. Symbols represent the densities of points across 50 independent replicates. Colors indicate different simulation settings (see Table 1 in main text for details). (TIF) [file pcbi.1003457.s008.tif]

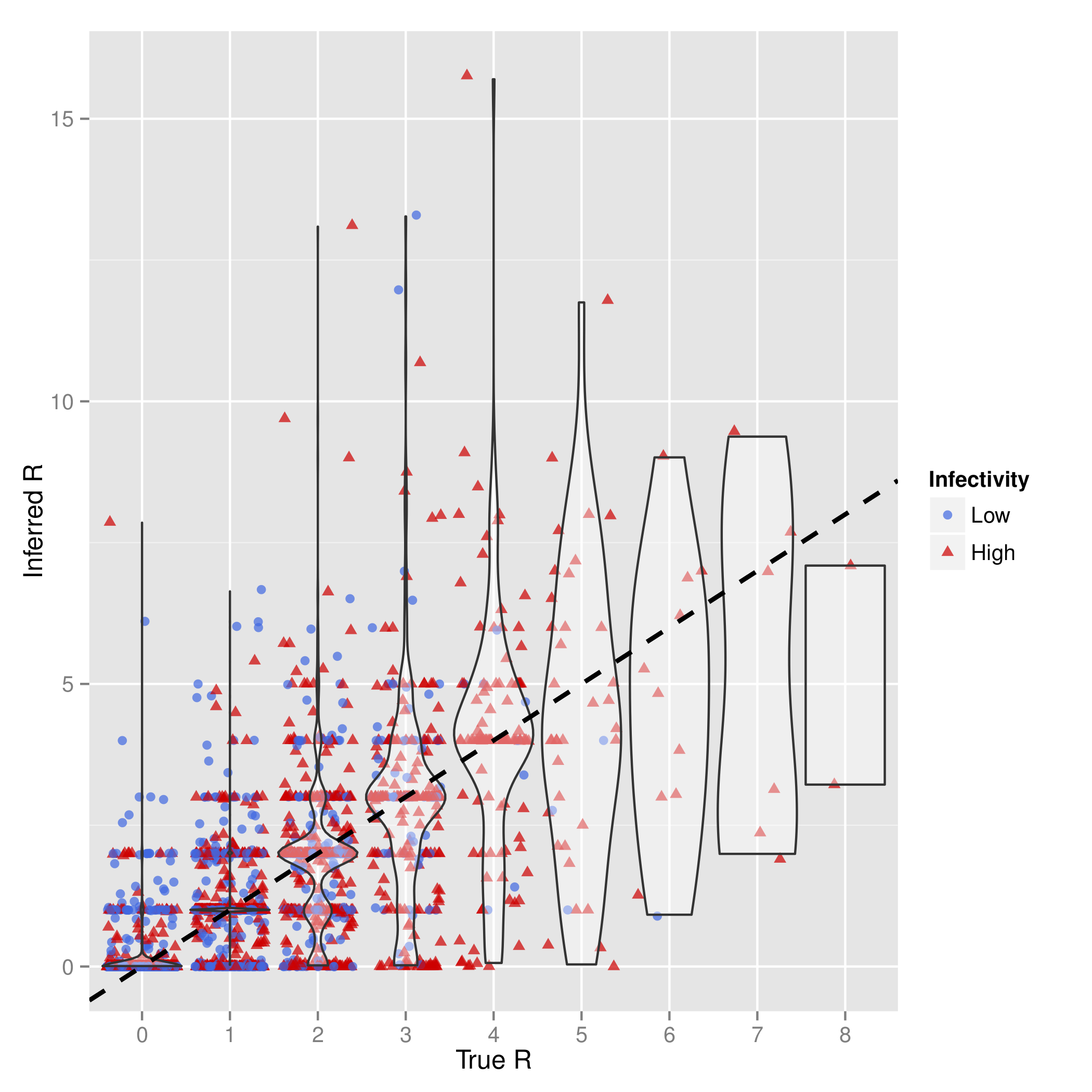

Supplement: Figure S6 — Inference of individual R with group-structured infectivity, using genetic information. This violinplot shows the estimates of individual effective reproduction numbers (R) for outbreaks incorporating group-structured infectivity. Results are based on 50 replicates. Densities represent individuals from both groups, while colored symbols (circles, crosses) distinguish the groups. The dashed line indicates identity. (TIF) [file pcbi.1003457.s009.tif]

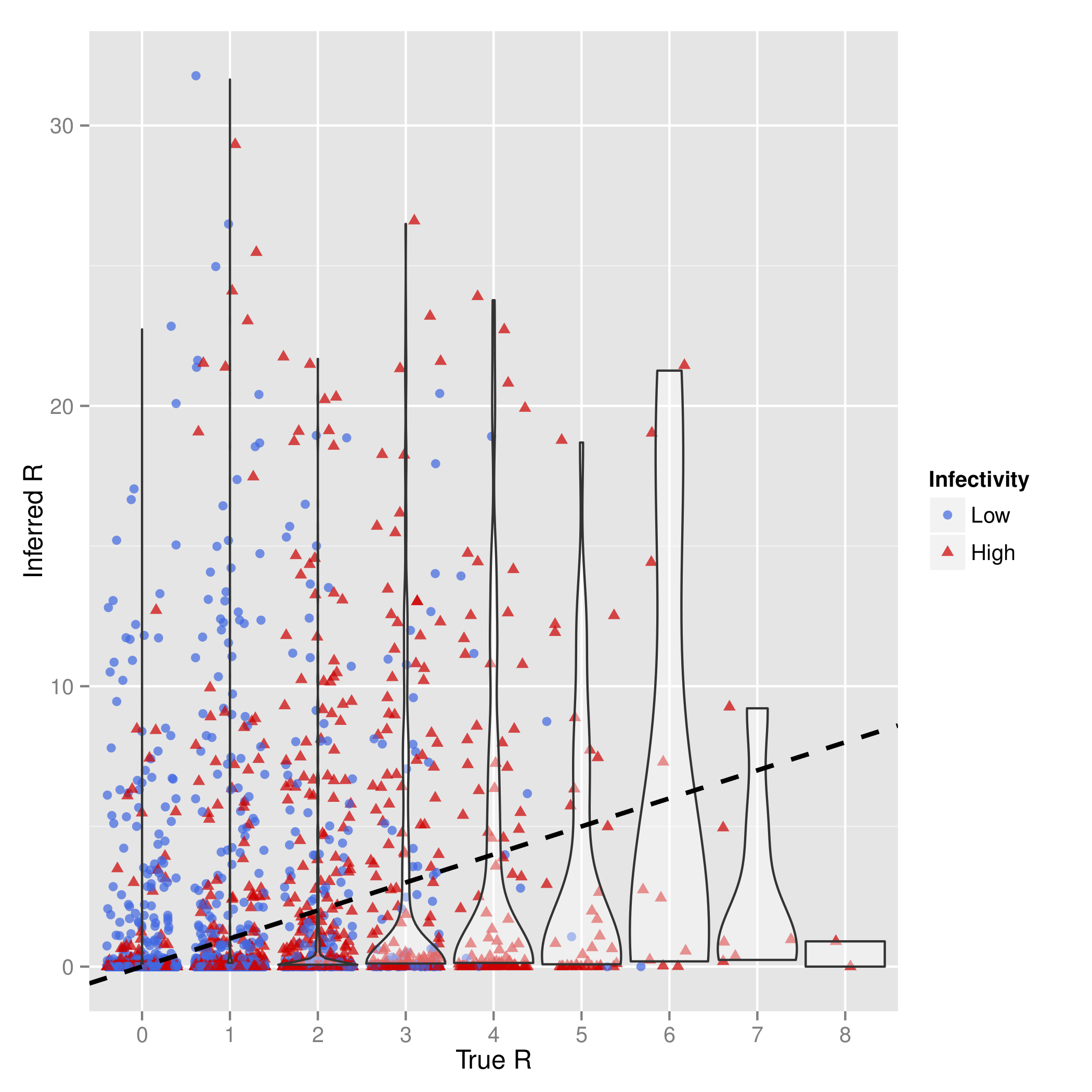

Supplement: Figure S7 — Inference of individual R with group-structured infectivity, without genetic information. This violinplot shows the estimates of individual effective reproduction numbers (R) for outbreaks incorporating group-structured infectivity. Results are based on 50 replicates, without the use of genetic information. Densities represent individuals from both groups, while colored symbols (circles, crosses) distinguish the groups. The dashed line indicates identity. (TIF) [file pcbi.1003457.s010.tif]

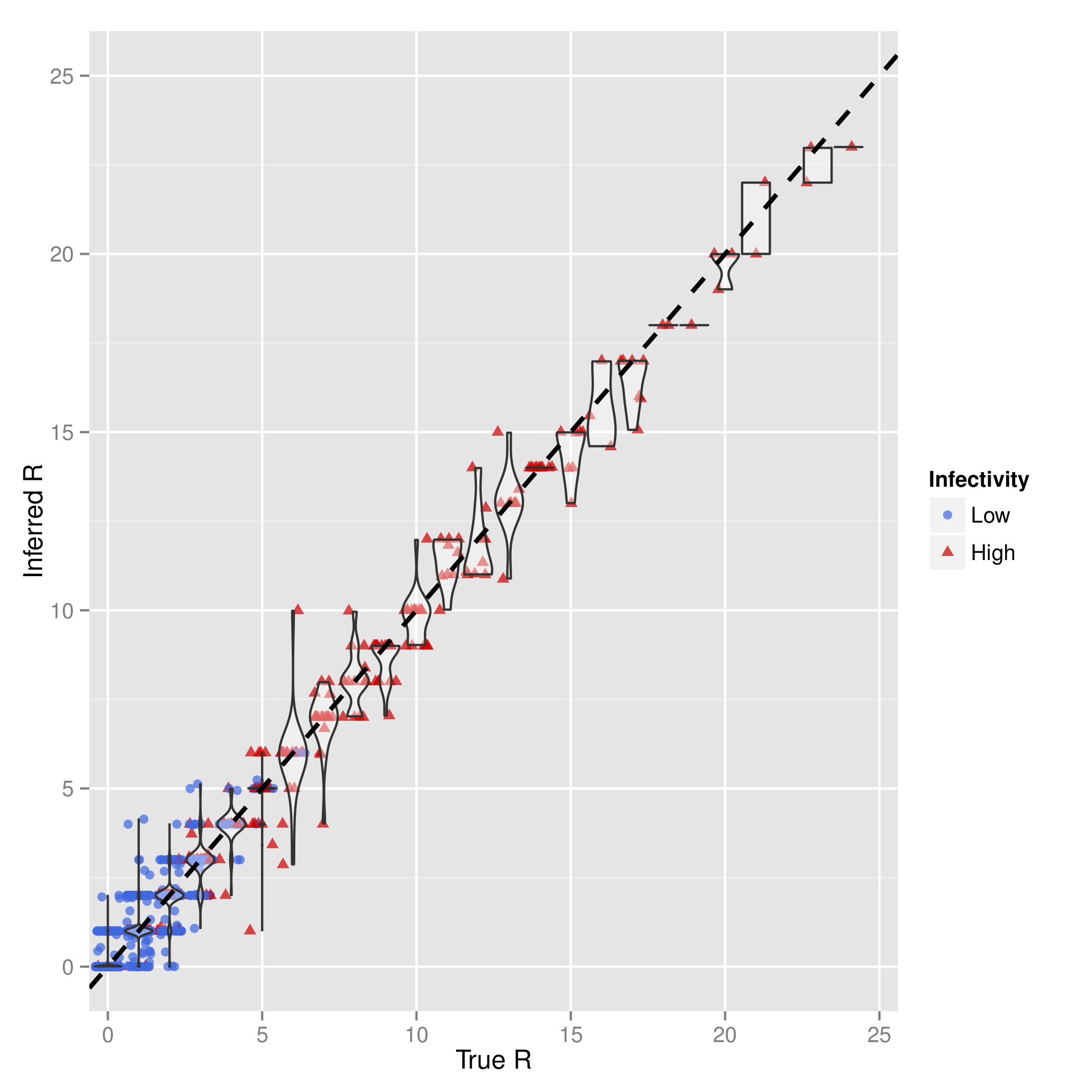

Supplement: Figure S8 — Inference of individual R in presence of super-spreaders, using genetic information. This violinplot shows the estimates of individual effective reproduction numbers (R) for outbreaks incorporating super-spreaders. Results are based on 50 replicates, without the use of genetic information. Densities represent all individuals, while colored symbols (circles, crosses) distinguish the super-spreaders from ‘normal’ individuals. The dashed line indicates identity. (TIF) [file pcbi.1003457.s011.tif]

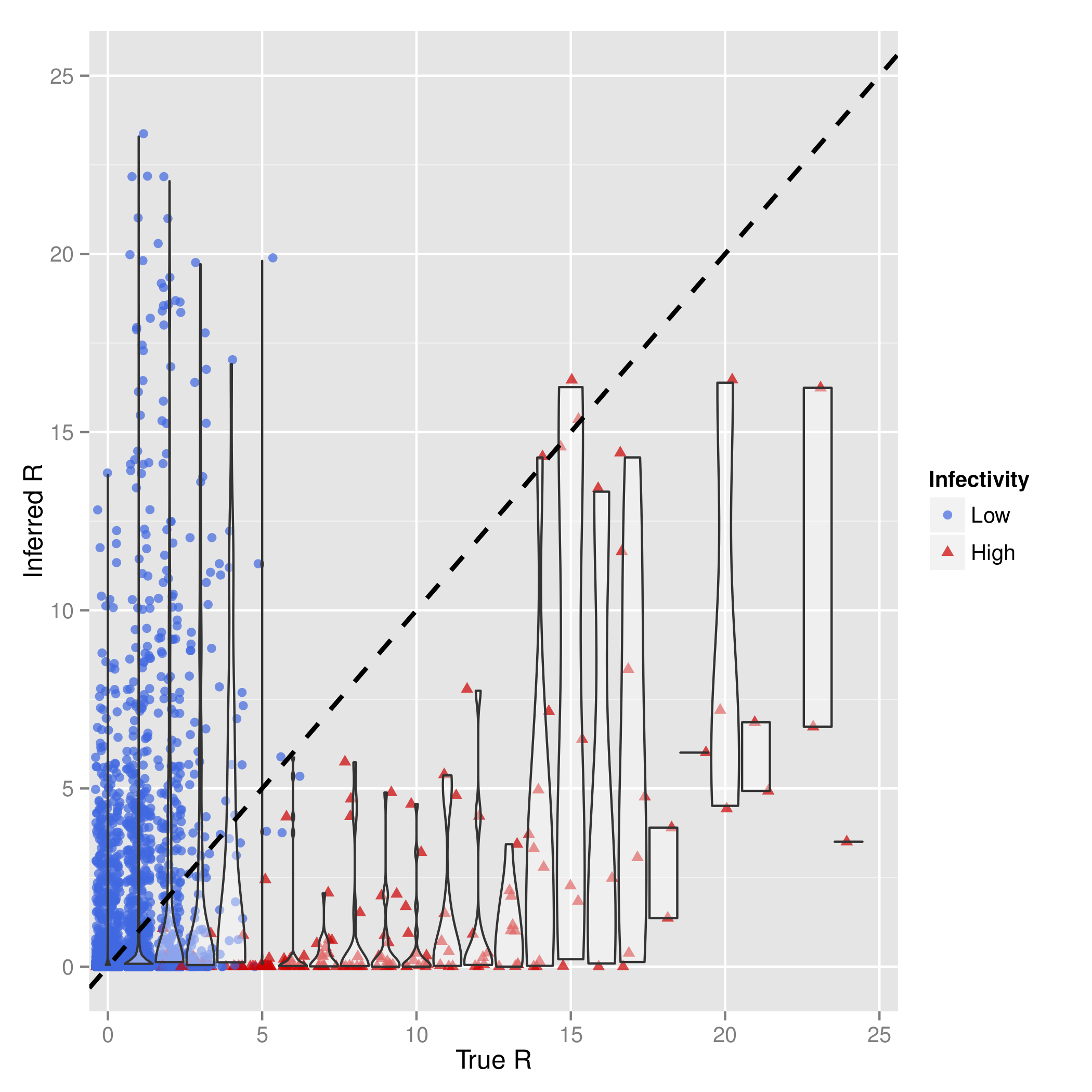

Supplement: Figure S9 — Inference of individual R in presence of super-spreaders, without genetic information. This violinplot shows the estimates of individual effective reproduction numbers (R) for outbreaks incorporating super-spreaders. Results are based on 50 replicates. Densities represent all individuals, while colored symbols (circles, crosses) distinguish the super-spreaders from ‘normal’ individuals. The dashed line indicates identity. (TIF) [file pcbi.1003457.s012.tif]

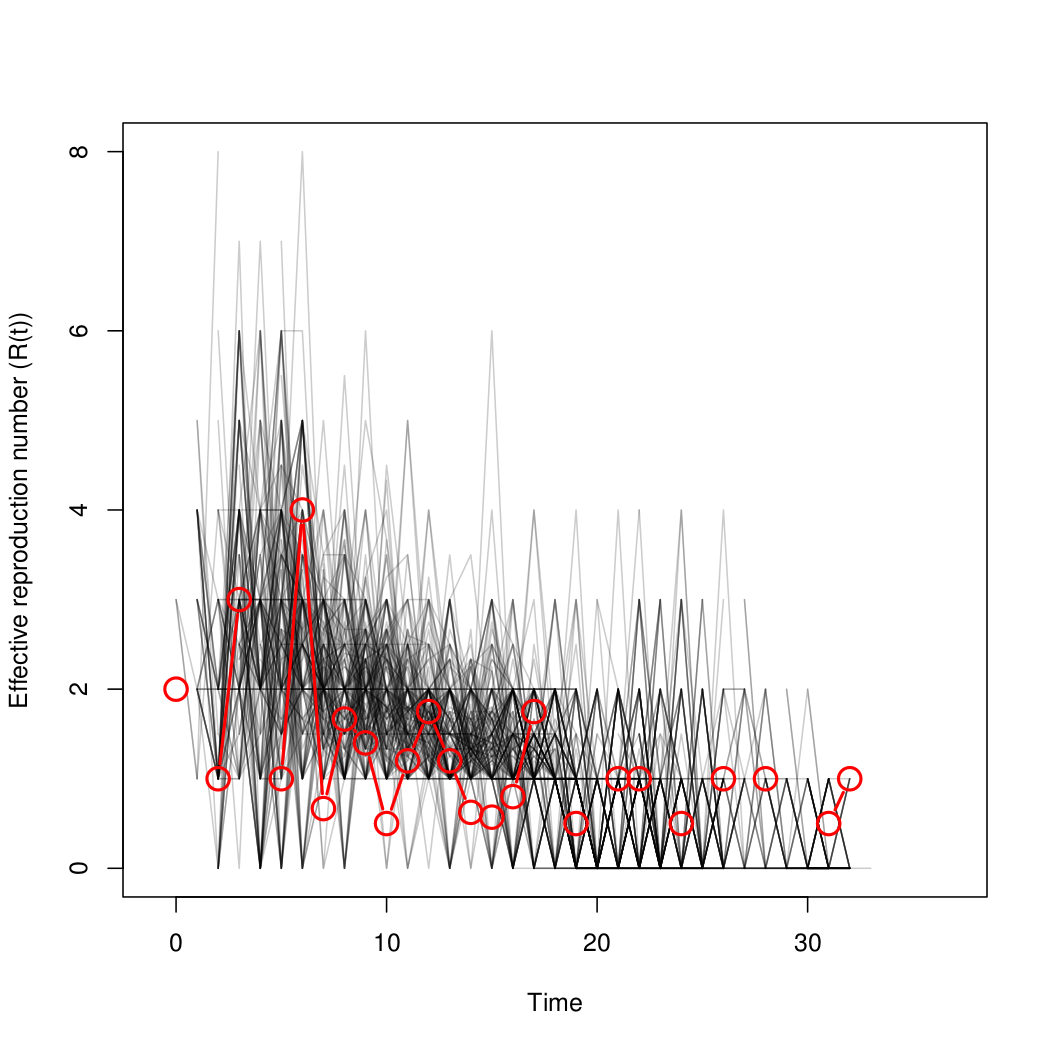

Supplement: Figure S10 — Example of reconstruction of the average effective reproduction number over time. This figure illustrates the inference of R over time in one simulation (setting ‘base’) derived from posterior ancestries. The actual values of R are shown in red. Missing values correspond to time steps without new infections. (TIF) [file pcbi.1003457.s013.tif]

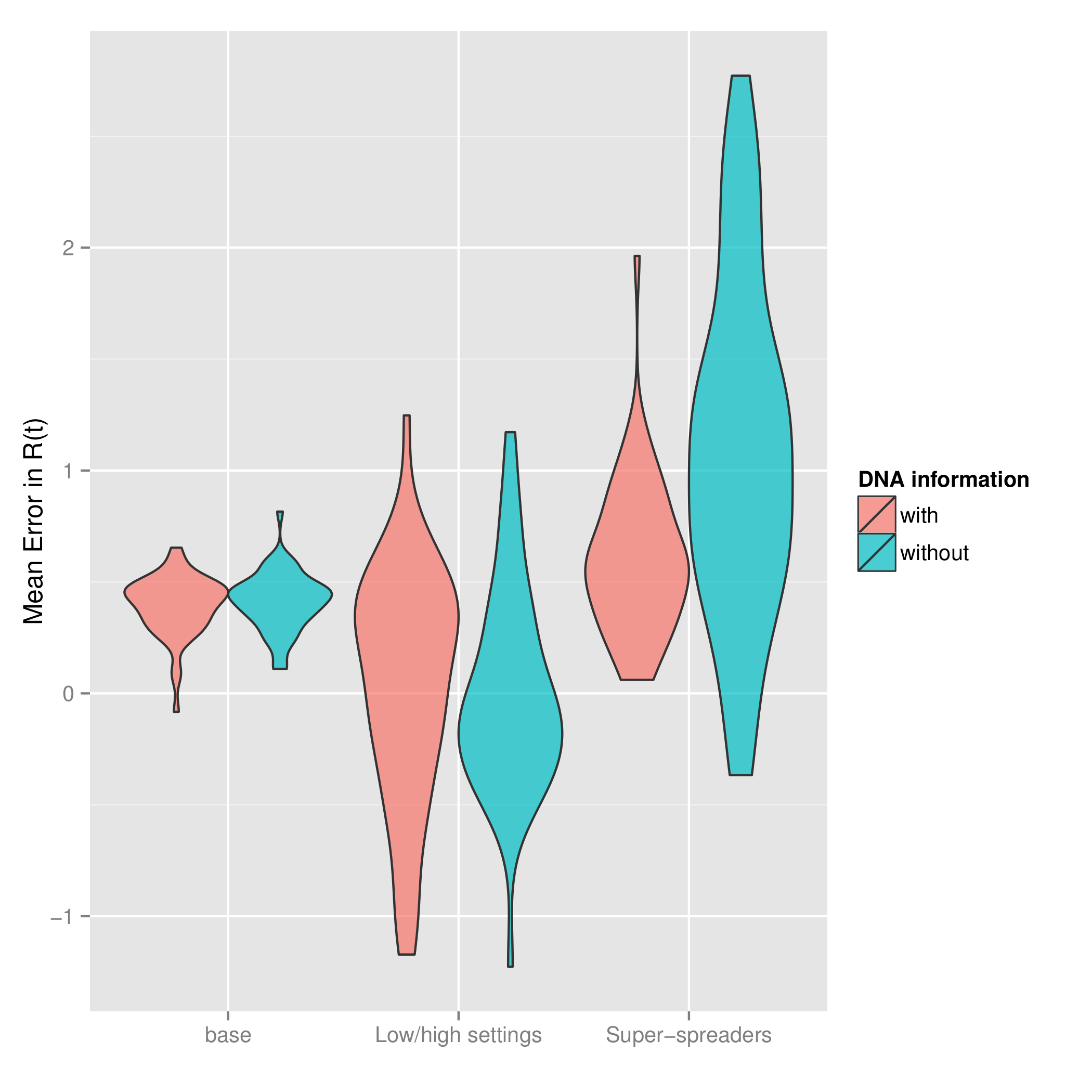

Supplement: Figure S11 — Inference of the average effective reproduction number over time. This violinplot shows the mean error (ME) in the estimated values of R over time, in basic simulated outbreaks (setting ‘base’), and in outbreaks incorporating group-structured infectivity (‘Low/high settings’) or super-spreaders (‘Super-spreaders’). Each box represents 50 independent replicates. (TIF) [file pcbi.1003457.s014.tif]

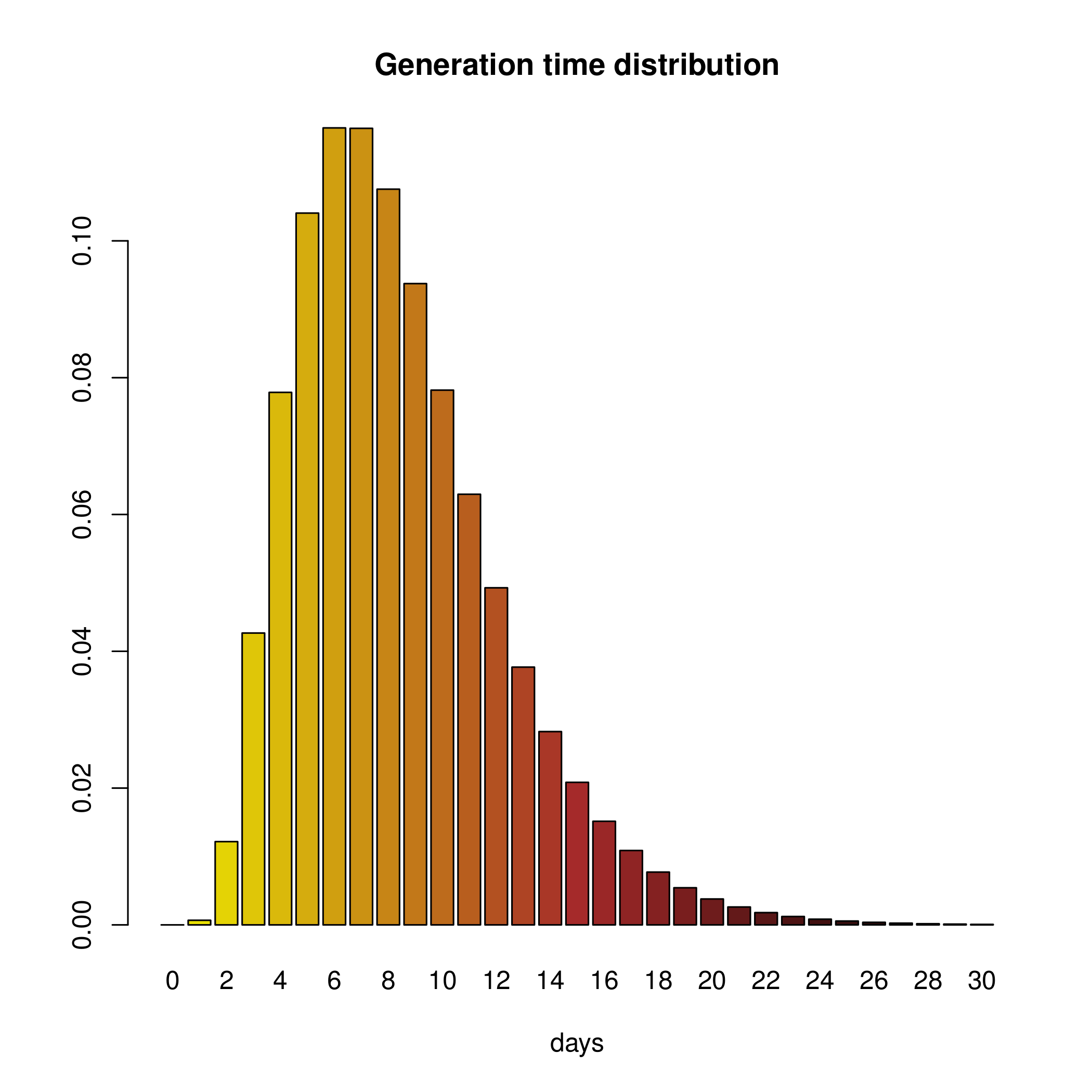

Supplement: Figure S12 — Generation time distribution for SARS. Probability mass function of the time between primary and secondary cases (i.e., time after which a newly infected individual creates new infections). (TIF) [file pcbi.1003457.s015.tif]

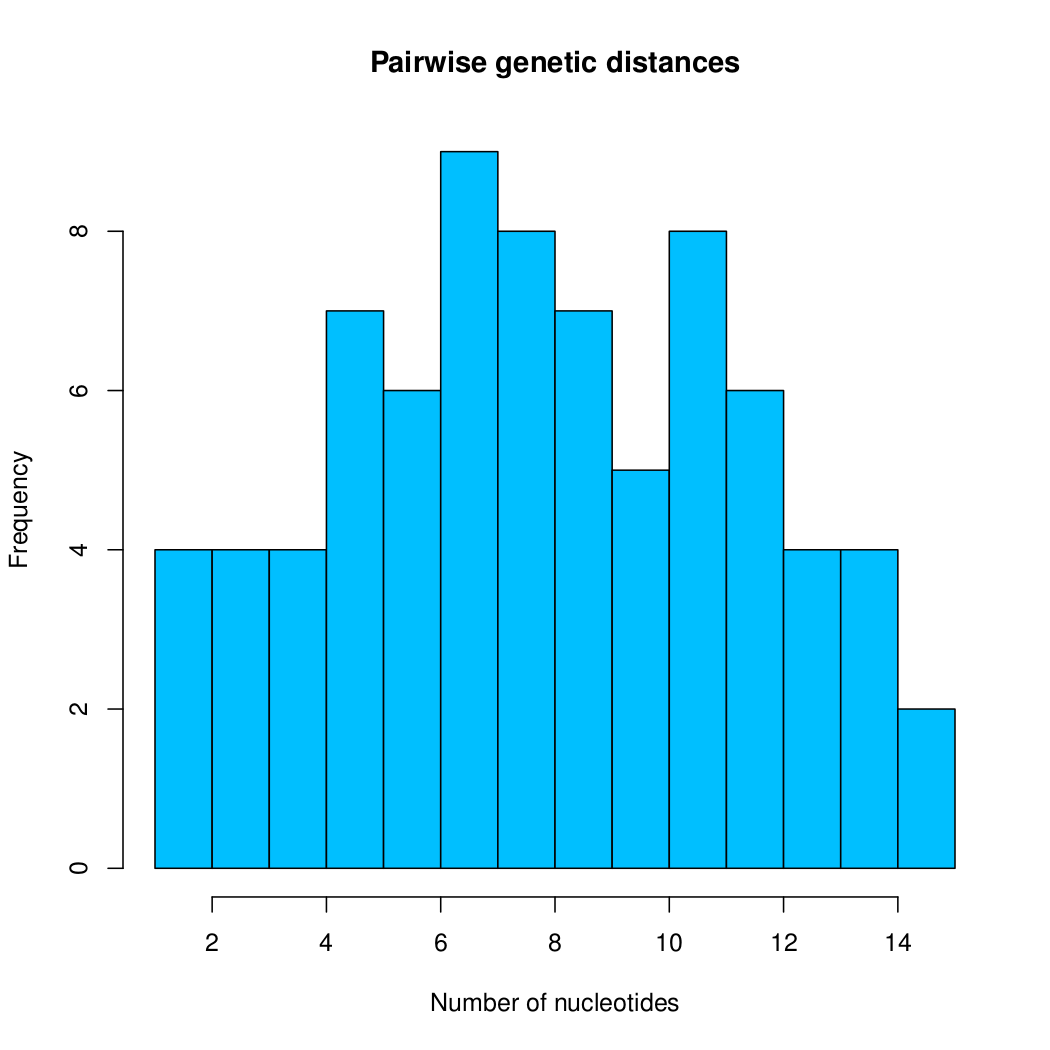

Supplement: Figure S13 — Distribution of pairwise genetic distances in the SARS data. This histogram shows the distribution of the pairwise distances between the 13 SARS genomes of the 2003 Singapore outbreak, expressed in number of differing nucleotides. (TIF) [file pcbi.1003457.s016.tif]

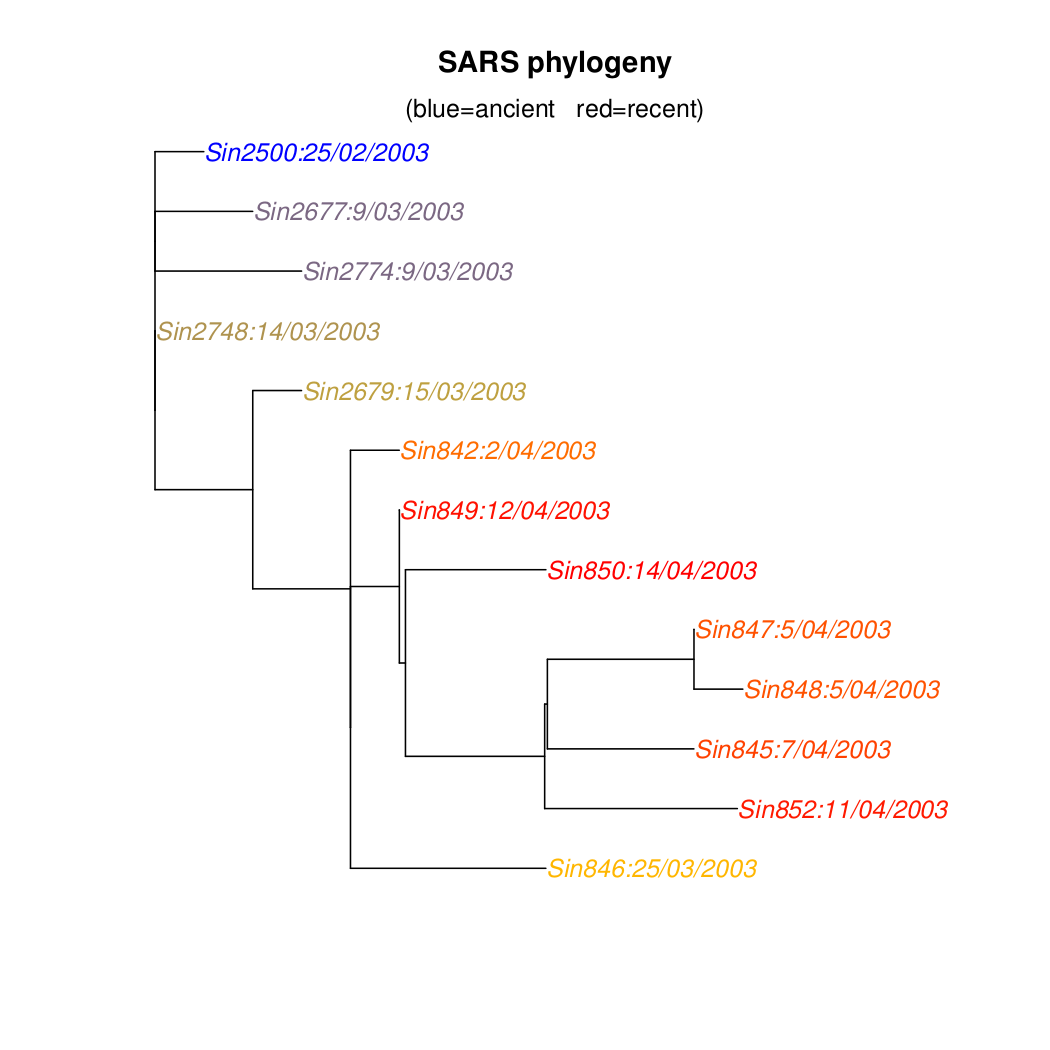

Supplement: Figure S14 — Phylogenetic tree of the SARS data. Neighbor-Joining tree based on the Hamming distances (see Fig. S10) between the 13 SARS genomes of the 2003 Singapore outbreak. The tree is rooted to the most ancient isolate (Sin2500). Colors indicate time, with more ancient isolates in blue and more recent isolates in red. This tree was realized using the package ape for the R software. (TIF) [file pcbi.1003457.s017.tif]

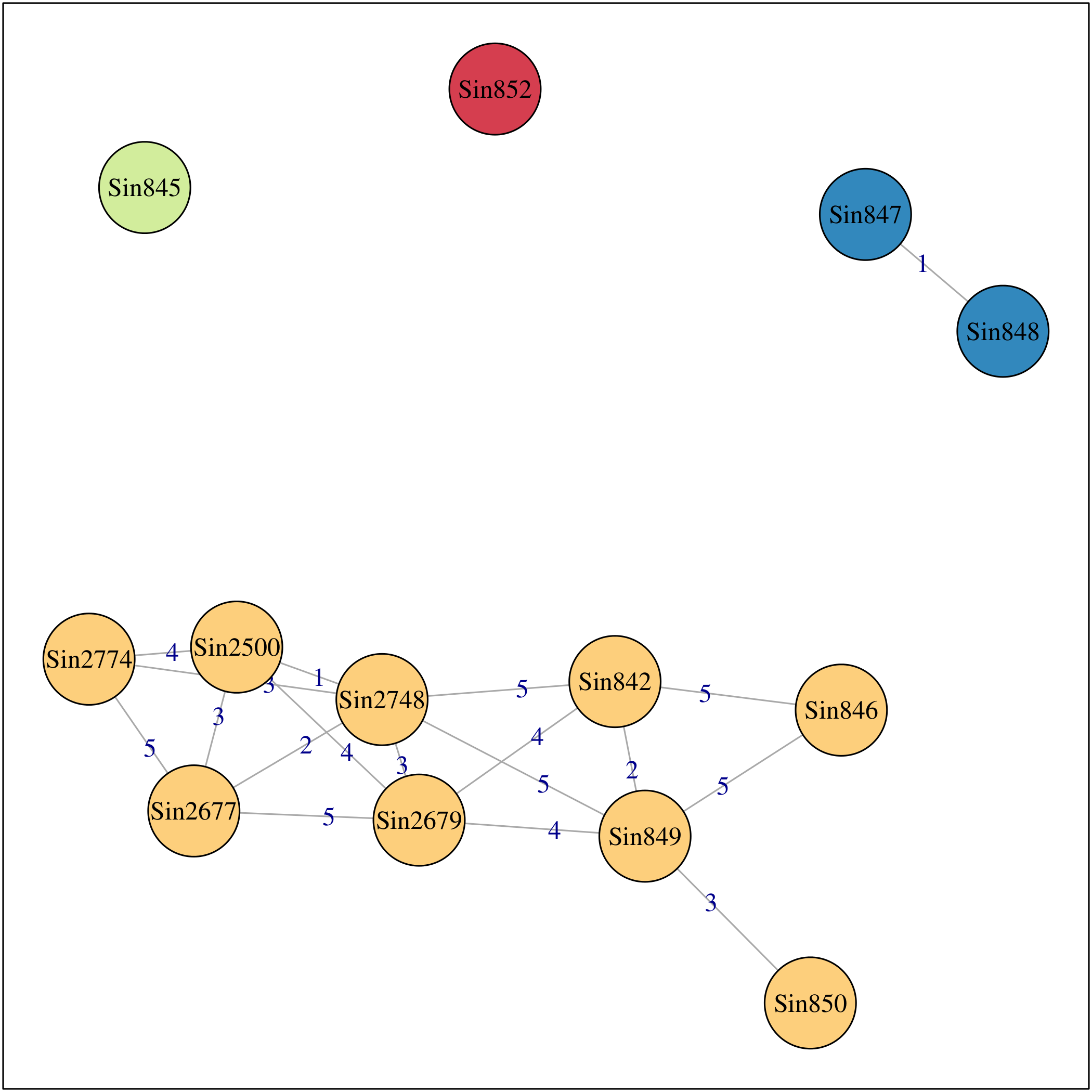

Supplement: Figure S15 — Graph connecting closely related genomes. These clusters were defined using a graph approach where pairs of genomes are connected when they are distant by no more than 5 mutations from each other (function ‘gengraph’ from the R package adegenet). The resulting connected components form clusters represented using different colors. Numbers annotating the edges represent the number of mutations between pairs of genomes. For the sake of readability, the dates were removed from the labels of the sequences. (TIF) [file pcbi.1003457.s018.tif]

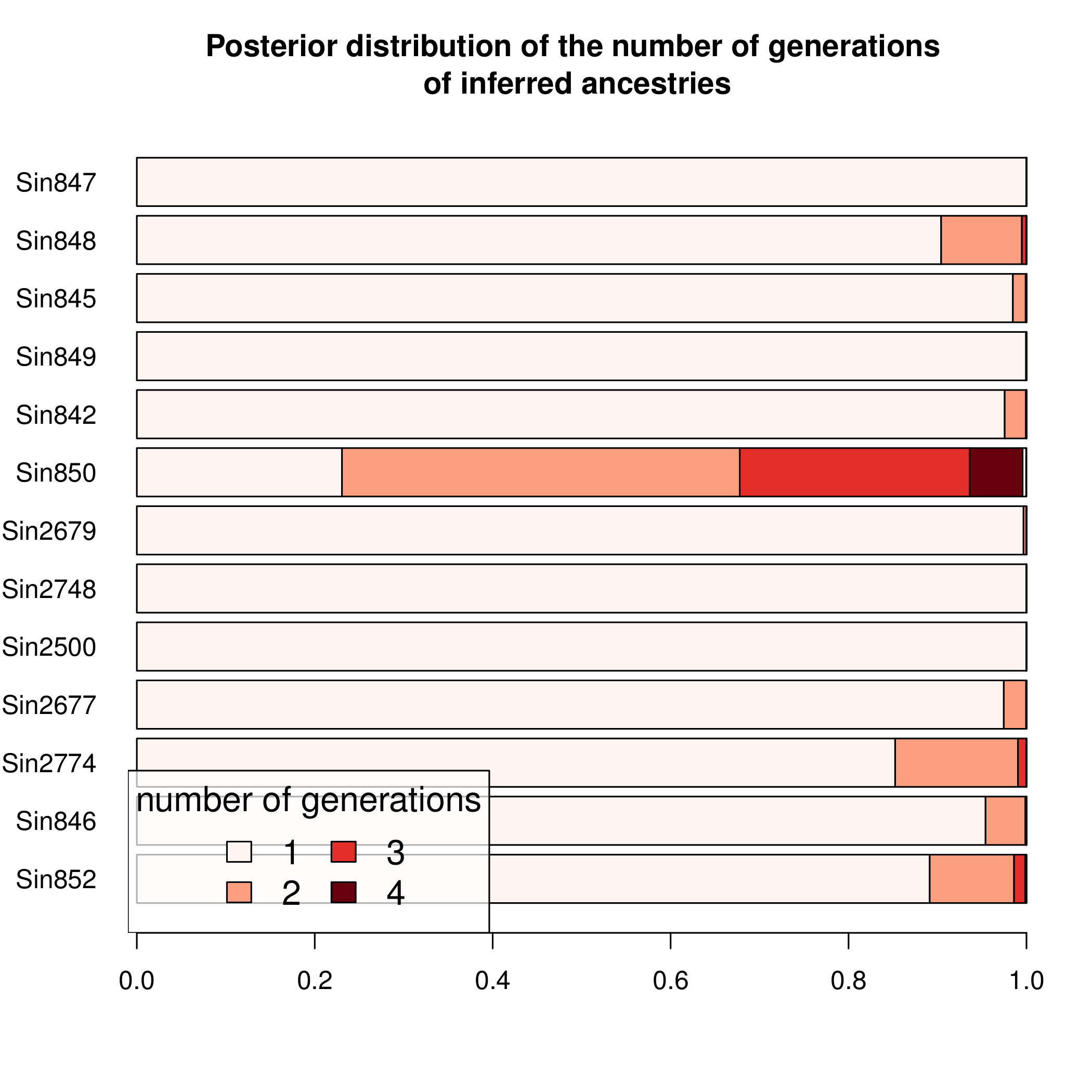

Supplement: Figure S17 — Number of generation of the inferred ancestries in SARS data. This barplot represents the posterior distribution of the number of generations in inferred ancestries for each case (rows). (TIF) [file pcbi.1003457.s020.tif]

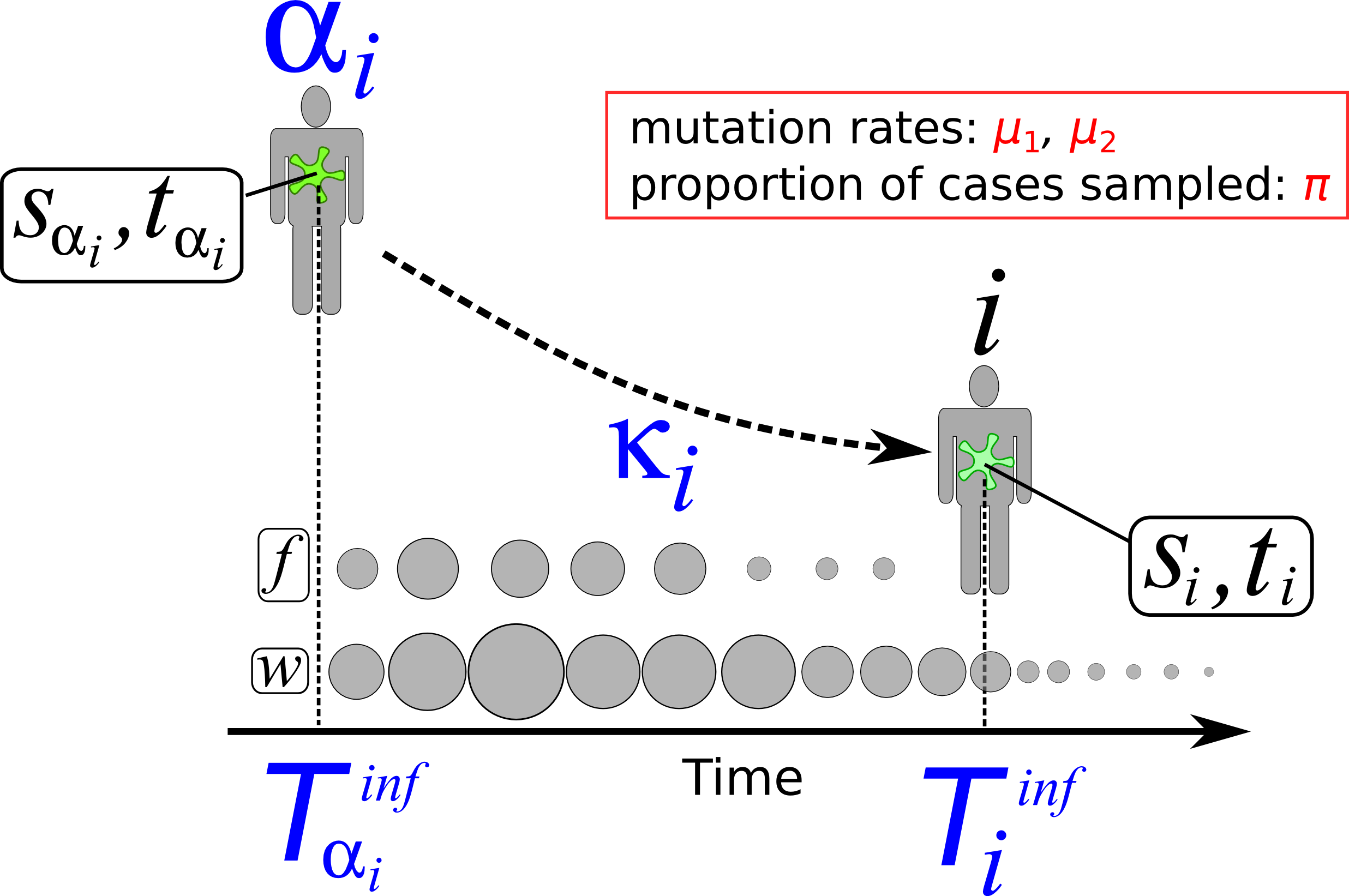

Supplement: Figure S18 — Outline of the transmission model. This diagram illustrates the concepts and notations used in the transmission model, using a single transmission event. Data are represented in black, augmented data in blue, and parameters in red. For both time interval distributions (w and f), larger circles are used to indicate larger probabilities. (TIF) [file pcbi.1003457.s021.tif]

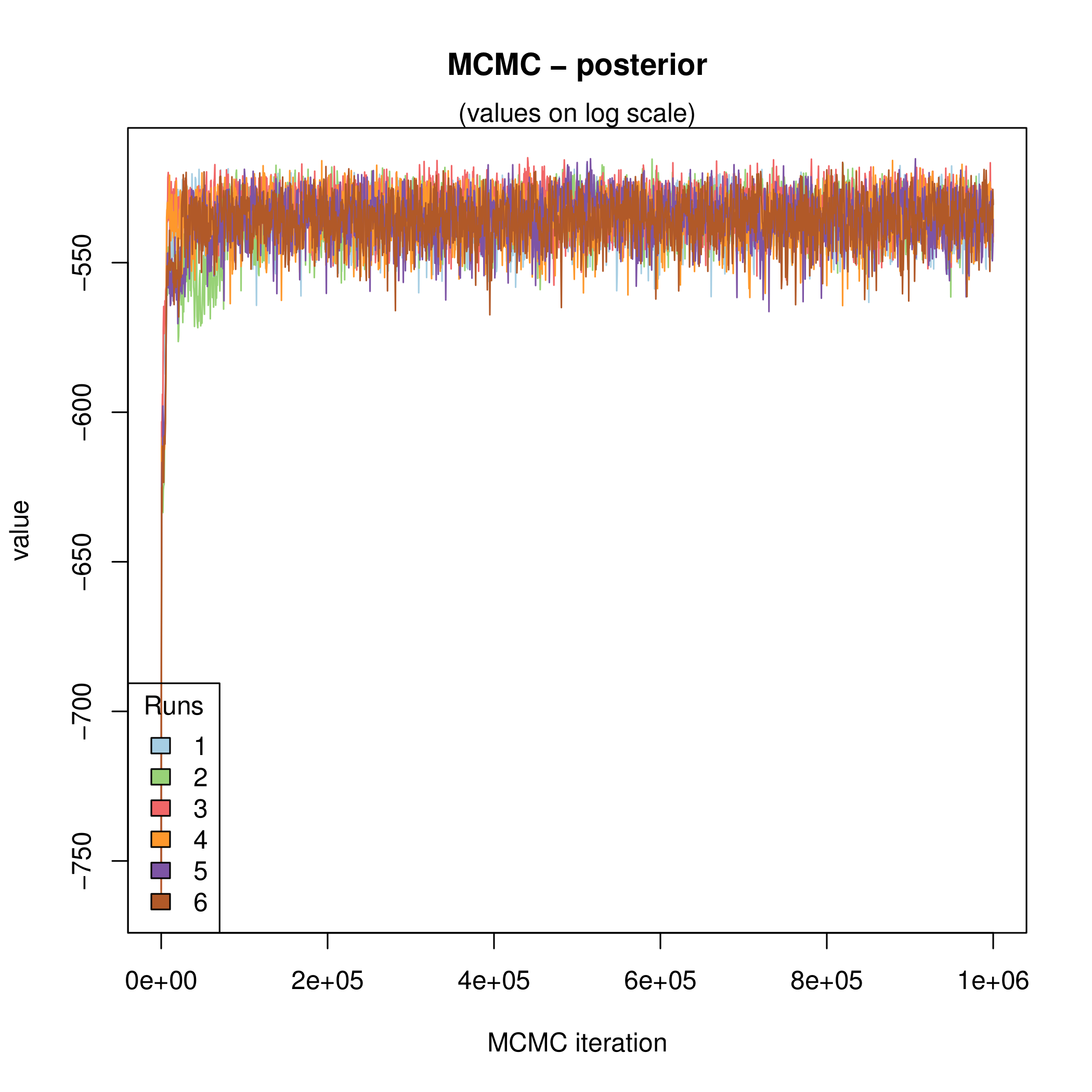

Supplement: Figure S19 — Convergence of the MCMC for the analysis of SARS data. This figure shows the posterior values of 6 independent MCMC (1,000,000 iterations each) used for the analysis of the SARS data. The burnin period chosen visually was 100,000 iterations. (TIF) [file pcbi.1003457.s022.tif]
