# Supplementary material for: Bayesian Reconstruction of Disease Outbreaks by Combining Epidemiologic and Genomic Data
Source: PLoS Comput Biol. 2014 Jan 23;10(1):e1003457. doi: 10.1371/journal.pcbi.1003457 (PMC3900386; doi:10.1371/journal.pcbi.1003457)
Supplement: Text S1 — Supporting methods and tables. This file describes the priors and parameter estimation procedures used in outbreaker, as well as the settings used in the SARS outbreak analysis and the supporting table S1. (PDF) [file pcbi.1003457.s023.pdf]

SUPPORTING INFORMATION

Bayesian reconstruction of disease outbreaks by combining epidemiologic and genomic data

Thibaut Jombart, Anne Cori, Xavier Didelot, Simon Cauchemez,
Christophe Fraser, Neil Ferguson

SUPPORTING METHODS

Description of the priors

The generation time distribution w and the parameters are given independent priors, so that:

$$p(\mu, \pi) = p(\mu)p(\pi)$$

We assume that little prior information is available for the mutation rate so that $p(\mu)$ is a uniform distribution on [0,1]. $p(\pi)$ is the probability density function of a Beta distribution with fixed, user-defined parameters, which provides a flexible way of indicating possible knowledge on sampling coverage. By default, parameters of this distribution are 10 and 1, corresponding to a dense coverage of the outbreak.

Parameter estimation

A Markov Chain Monte Carlo (MCMC) approach is used to draw samples from the joint posterior distribution (1). Metropolis-Hastings algorithm [2] is used to explore the augmented data and parameter space. For μ, and π, the variance of each proposal distribution is first tuned until the acceptance rates lies between 25% and 50%. The corresponding iterations are automatically discarded from the retained samples. The burn-in period is assessed by examining visually the convergence of the chains (using the function *plotChains* in outbreaker). By default, the burn-in period is set to 20,000 iterations.

Settings of *outbreaker* for the analysis of the 2003 SARS outbreak in Singapore

Data were analyzed using the parallelized implementation of the model (function *outbreaker.parallel*) with 6 independent runs, a chain length of 1,000,000 iterations, a flat prior for π and initializing the algorithm using a star-like tree (the index case being the infector of all other cases). The burnin period was assessed visually and set conservatively to 100,000 iterations (Fig. S19). All results reported are based on posterior samples merged across runs after discarding burnin periods (Fig. S19).

SUPPORTING TABLES

Table 1: simulation results. All results are based on runs of *outbreaker* without prior information, all parameters being estimated. Each row corresponds to a different simulation setting (please refer to Table 2, main text, for further description). Values indicate averages over replicates, with 95% credibility intervals indicated between square brackets.

	**Proportion of correct ancestry***	**Mean support for true ancestor****	**Mean support for true kappa****	**Mean absolute infection date error****	**Proportion of imported case detected**	**Mean absolute error in μ****	**Mean absolute error in π*****
**Base**	0.82 [0.7;0.92]	0.81 [0.7;0.9]	0.99 [0.95;1]	0.94 [0.77;1.08]	0.82 [0;1]	0.17 [0.02;0.44]	0.03 [0.01;0.15]
**No import**	0.82 [0.72;0.93]	0.81 [0.71;0.91]	0.99 [0.96;1]	0.94 [0.78;1.11]	NA	0.19 [0.02;0.5]	0.02 [0.01;0.09]
**Many imports**	0.78 [0.67;0.87]	0.8 [0.72;0.9]	0.95 [0.85;1]	0.94 [0.85;1.06]	0.44 [0.06;1]	0.3 [0.03;0.78]	0.1 [0.01;0.37]
**No mutation**	0.11 [0.06;0.23]	0.08 [0.05;0.2]	0.92 [0.83;0.98]	0.95 [0.86;1.09]	0.05 [0;0.5]	NA	0.08 [0.03;0.18]
**Fast evolution**	0.91 [0.83;1]	0.92 [0.84;1]	0.99 [0.94;1]	0.93 [0.78;1.04]	0.85 [0;1]	0.2 [0.02;0.41]	0.03 [0.01;0.11]
**Low R**	0.83 [0.54;1]	0.83 [0.57;0.99]	0.97 [0.91;1]	0.91 [0.65;1.15]	0.77 [0;1]	0.24 [0.02;0.85]	0.06 [0.02;0.23]
**High R**	0.73 [0.59;0.85]	0.72 [0.57;0.84]	1 [0.99;1]	0.92 [0.82;1.05]	0.94 [0;1]	0.19 [0.02;0.57]	0.01 [0.01;0.01]
**75% missing cases**	0.61 [0.4;0.87]	0.7 [0.4;1]	0.42 [0.18;0.72]	0.96 [0.7;1.21]	0.04 [0;0.33]	0.77 [0.18;1.62]	0.39 [0.16;0.63]
**50% missing**	0.72 [0.53;0.84]	0.75 [0.6;0.88]	0.56 [0.42;0.7]	0.91 [0.73;1.12]	0.24 [0;1]	0.53 [0.1;1.1]	0.32 [0.15;0.46]

**cases**							
**25% missing cases**	0.77 [0.66;0.88]	0.78 [0.69;0.93]	0.75 [0.63;0.83]	0.91 [0.73;1.11]	0.54 [0;1]	0.3 [0.04;0.68]	0.17 [0.01;0.24]
**Short generation**	0.7 [0.58;0.81]	0.68 [0.55;0.78]	0.99 [0.95;1]	0.39 [0.24;0.57]	0.97 [0.67;1]	0.14 [0.02;0.36]	0.02 [0.01;0.05]
**Long generation**	0.85 [0.75;0.94]	0.89 [0.77;0.98]	0.89 [0.84;0.95]	4.47 [4.09;5.02]	0.01 [0;0.19]	0.35 [0.09;0.81]	0.17 [0.08;0.31]

* based on consensus tree

** based on the entire posterior, mutation rate re-estimated in number of mutations per unit of time from the posterior trees

*** based on the entire posterior
